# Quantification of esterified oxylipins following HILIC-fractionation of lipid classes

**DOI:** 10.1016/j.jlr.2025.100950

**Published:** 2025-11-21

**Authors:** Luca M. Wende, Laura Carpanedo, Lilli Scholz, Nadja Kampschulte, Annette L. West, Philip C. Calder, Nils Helge Schebb

**Affiliations:** 1 Food Chemistry, School of Mathematics and Natural Sciences, University of Wuppertal, Wuppertal, Germany; 2 School of Human Development and Health, Faculty of Medicine, University of Southampton, Southampton, UK; 3 NIHR Southampton Biomedical Research Centre, University Hospital Southampton NHS Foundation Trust and University of Southampton, Southampton, UK

**Keywords:** oxidized lipids, solid-phase extraction, hydrophilic interaction liquid chromatography, lipidomics, glycerophospholipids, omega-3 fatty acids, fish oil, human plasma, nutrition, esterified oxylipins

## Abstract

Several oxylipins are lipid mediators derived from the oxidation of polyunsaturated fatty acids (PUFAs). The majority of oxylipins in biological samples occurs esterified in neutral lipids (nLs) and phospholipids (PLs). They are commonly quantified indirectly following alkaline hydrolysis, providing excellent sensitivity. However, the information in which lipid classes the oxylipins occur is lost. The direct analysis of oxidized lipids is currently not sensitive enough to detect all esterified oxylipins. Here, a new hydrophilic interaction liquid chromatography (HILIC)-based lipid class fractionation using solid-phase extraction (SPE) cartridges was developed, separating lipids into nLs and 4 PL fractions using a single column. Esterified oxylipins in the fractions were quantified following alkaline hydrolysis to sensitively pinpoint in which lipid classes they are bound in plasma. The fractionation was extensively characterized for different lipid extracts, demonstrating high separation efficiency and recovery using labeled standards and untargeted analysis of endogenous lipids. Esterified oxylipins in the fractions were quantitatively detected. Based on the results from two independent human plasma pools, including SRM 1950, it is shown that: hydroxy-linoleic acids and hydroxy-α-linolenic acids are preferably bound to nLs, whereas long-chain hydroxy-PUFAs and PUFAs (i.e. ARA EPA and DHA) are predominantly esterified to phospholipid classes. Supplementation of n3-PUFAs for 12 months led to an increase in EPA- and DHA-derived oxylipins in all lipid fractions, with the highest increase in hydroxy-PUFAs in nLs. This demonstrates a precursor PUFA-dependent binding of oxylipins and a direct effect of diet on esterified oxylipins in plasma.

## Graphical abstract

**Figure:**
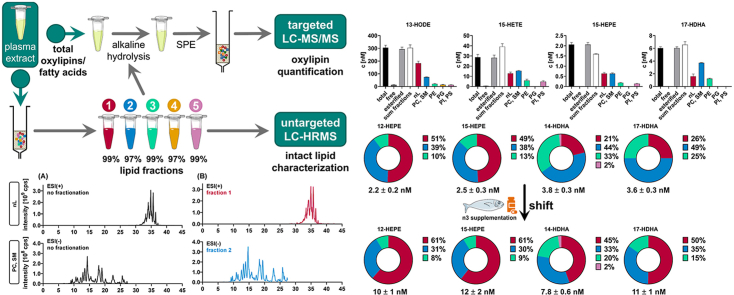

Several eicosanoids and other oxylipins are potent lipid mediators derived from the oxidation of PUFAs (1). They can be formed either nonenzymatically by radical-mediated and stereo-random (aut)oxidation or enzymatically by different enzymes, leading to position- and stereo-specific products (2). The enzymatic pathways of formation play important roles in the regulation of inflammation, immunity, thrombosis and other biological responses (3). PUFAs such as ARA can be oxidized by enzymes including cyclooxygenases (COX), lipoxygenases (LOX), and cytochrome P450 monooxygenases (CYP) (4). LOX enzymes (e.g., 5-LOX, 12-LOX, 15-LOX-1, 15-LOX-2) form regio- and stereoselective hydroperoxy-PUFAs at different positions, which can be reduced to hydroxy-PUFAs by enzymes such as glutathione peroxidases (5, 6). The oxidation of PUFAs by CYP results in the formation of terminal hydroxy-PUFAs and/or cis-(*R*,*S* and *S*,*R*)-epoxy-PUFAs (7). Oxylipins can be formed from n6-PUFAs such as ARA and n3-PUFAs, including EPA and DHA, giving rise to a large number of structurally diverse oxidized fatty acids (8).

The vast majority of oxylipins, i.e. epoxy-PUFAs and hydroxy-PUFAs present in blood plasma (9), serum (10), cells (11), and tissues (12) occur in esterified form. Similar to their PUFA precursors, oxylipins are bound as esters in lipids such as cholesterol esters (Chol Esters), triacylglycerols (TGs), and polar lipids, i.e., glycerophospholipids (PLs) (13). Indeed, >90% of the hydroxy-PUFAs present in plasma are esterified (14). Little is known about which lipids the oxylipins are esterified in but some studies suggest that they are predominantly bound to TGs and PLs (15). Notably, oxylipins such as 18:2;OH, and 20:4;OH were found esterified in PC, TG, and Chol Ester species in human plasma (16), as well as in PC species in human serum (17).

Analysis of esterified oxylipins is challenging due to their low abundances in biological samples. Esterified oxylipins can be analyzed directly as oxidized PLs or TGs by LC-MS, providing detailed information about the binding form and lipid species of the bound oxylipin (13, 18). Quantitative targeted LC-MS/MS of oxPLs (bearing oxylipins) showed a distinct incorporation of hydroxy-PUFAs in both, oxidized PL classes and species, in human cells, but such research has been limited to a few oxylipins bound in selected oxidized PLs (11, 19, 20).

Even targeted methods (19, 20) using the most sensitive instrumentation available are limited by the sensitivity because the low concentrations of oxylipins are distributed over a large number of lipid classes and species including neutral lipids (nLs) such as TG, Chol Ester and PL classes such as phosphatidyl-choline (PC), -ethanolamine (PE), -glycerol (PG), -inositol (PI), and -serine (PS). This is highlighted in (Fig. 1), demonstrating, as an example for 15-HETE, the vast number of possible binding forms. Moreover, the direct analysis of esterified oxylipins is limited by the lack of available standards, hindering identification and quantification (11, 16, 21). Thus, quantification of esterified oxylipins in biological samples is commonly performed indirectly by alkaline hydrolysis followed by quantification of the resulting free oxylipins using established targeted LC-MS/MS methods (10, 14, 22, 23, 24). However, the information about which lipid class the oxylipins are bound to gets lost in the saponification step.

**Fig. 1:**
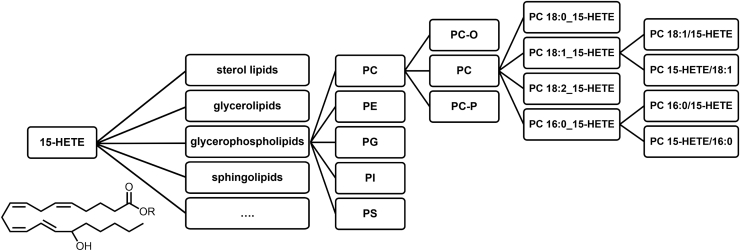
Distribution of esterified oxylipins across different lipid classes and species. This illustration highlights for 15-HETE and PLs the large number of different binding forms of esterified oxylipins.

To better understand in which lipid classes esterified oxylipins are bound, we developed a sensitive orthogonal approach: lipid class fractionation using a solid-phase extraction (SPE) cartridge followed by LC-MS/MS quantification of esterified oxylipins in the different lipid fractions after alkaline hydrolysis. Chromatographic fractionation of lipids using SPE cartridges is frequently applied, allowing the separation of Chol Esters, TGs, and different PLs such as PC, PE, PG, PI/PS classes (25, 26, 27). However, for oxylipins, this was only used so far to differentiate between nLs and PLs in cells (28) and plasma (29). Here, we utilize a not previously reported hydrophilic interaction liquid chromatography (HILIC) approach for separation requiring only a single SPE column to efficiently fractionate lipids in nLs and four different PL fractions. The performance of the fractionation was characterized for different biological samples using non-targeted lipidomics. The method was then applied to the investigation of esterified oxylipins in human plasma and the changes induced by n3-PUFA supplementation. A distinct PUFA-dependent binding of hydroxy-PUFAs in plasma lipids was unveiled.

## Materials and Methods

### Chemicals and biological material

The internal standard mixtures EquiSPLASH Lipidomix (330731-1EA) containing PC 15:0/18:1[D7], lyso-PC 18:1[D7]/0:0, PE 15:0/18:1[D7], lyso-PE 18:1[D7]/0:0, PG 15:0/18:1[D7], PI 15:0/18:1[D7], PS 15:0/18:1[D7], TG 15:0/18:1[D7]/15:0, diacylglycerol (DG) 15:0/18:1[D7]/0:0, monoacylglycerol (MG) 18:1[D7]/0:0/0:0, Chol Ester 18:1[D7], SM 18:1;O2/18:1[D9], C15 Ceramide (Cer)[D7] (each 100 μg/ml), and SPLASH Lipidomix (30707-1EA) containing PC 15:0/18:1[D7], lyso-PC 18:1[D7]/0:0, PE 15:0/18:1[D7], lyso-PE 18:1[D7]/0:0, PG 15:0/18:1[D7], PI 15:0/18:1[D7], PS 15:0/18:1[D7], TG 15:0/18:1[D7]/15:0, DG 15:0/18:1[D7]/0:0, MG 18:1[D7]/0:0/0:0, Chol Ester 18:1[D7], sphingomyelin (SM) 18:1;O2/18:1[D9], PA 15:0/18:1[D7], and free cholesterol[D7] (concentrations 1.8 μg/ml ‒ 329.1 μg/ml) were purchased from Avanti Polar Lipids (local supplier: Merck KGaA, Taufkirchen, Germany).

Acetonitrile (ACN) LC-MS grade, methanol (MeOH) LC-MS grade, 2-propanol (IPA) LC-MS grade, formic acid LC-MS grade, and acetic acid LC-MS grade were obtained from Fisher Scientific. Ultra-pure water was generated using a Barnstead Genpure Pro system from Thermo Fisher Scientific (Schwerte, Germany).

Human embryonic kidney 293 (HEK293) cells were obtained from the German Collection of Microorganisms and Cell Cultures GmbH (DSMZ, Braunschweig, Germany).

Citrated blood plasma samples (plasma pool 1) were generated with informed consent from three healthy adults approved by the ethics committee of the University of Wuppertal. In addition, human plasma reference material (National Institute of Standards and Technology (NIST) SRM 1950: Metabolites in Frozen Human Plasma) was analyzed.

### Cell culture

HEK293 cells were cultured in DMEM with high glucose (4.5 g/L), supplemented with 10% (v/v) FCS, 100 U/ml penicillin, 100 μg/ml streptomycin, and 1 mM sodium pyruvate. Culture occurred in a humidified atmosphere containing 5% CO_2_ at 37°C. Cells were seeded at a density of 5 × 10^6^ cells per 60.1 cm^2^ dish and harvested by scraping in ice-cold PBS after 24 h.

### Sample preparation

**Fig. 2:**
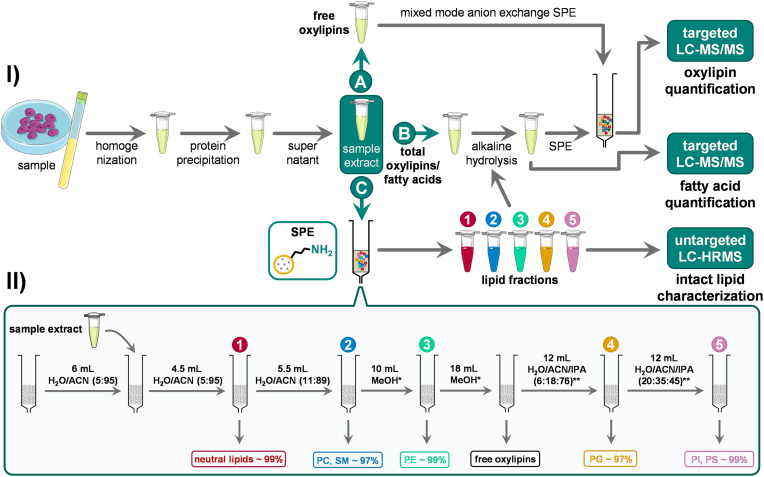
Scheme of the lipid class-specific analysis of esterified FA and oxylipins. I) Samples are homogenized, and proteins are precipitated. The supernatant is used for (A) the direct analysis of non-esterified oxylipins, (B) total oxylipins/FA after alkaline hydrolysis, and (C) total FA and oxylipin analysis following fractionation of lipid classes and alkaline hydrolysis. II) Lipid classes are fractionated by HILIC-based SPE into five fractions using a single column. The recovery of the lipids (determined for SPLASH ISs) in the fractions is indicated. ∗0.1% acetic acid; ∗∗20 mM ammonium formate + 0.1% formic acid.

As illustrated in Figure 2, 100 μl human plasma or 100 μl sonicated cell suspension (⁓400 μg cell protein) in H_2_O/MeOH (50:50, v/v) were transferred into a 1.5 ml microcentrifuge tube, followed by the addition of 10 μl of an internal standard (IS) mixture (EquiSPLASH for cell suspensions [2 μl/⁓400 μg cell protein], SPLASH Lipidomix for human plasma [5 μl/100 μl plasma], or deuterium-labeled oxylipins [each 1 pmol/100 μl plasma/⁓400 μg cell protein]). 10 μl of inhibitor/antioxidant solution (0.2 mg/ml butylated hydroxytoluene, 100 mM indomethacin, and 100 mM of the soluble epoxide hydrolase inhibitor trans-4-[4-(3-adamantan-1-yl-ureido)-cyclohexyloxy]-benzoic acid) was added. Protein precipitation was performed using 400 μl of ice-cold IPA, and samples were mixed and frozen at –80°C for at least 30 min. After centrifugation (20,000 *g*, 10 min, 4°C), the supernatant was used for SPE-based lipid class fractionation or quantification of oxylipins/FA without prior lipid fractionation.

### Fractionation of lipid classes using a solid-phase extraction cartridge

SPE for lipid class fractionation was performed using CHROMABOND NH_2_ cartridges (3 ml/500 mg sorbent per cartridge, particle size 45 μm; Machery-Nagel, Düren, Germany). Cartridges were washed with two volumes of MeOH and two volumes of ACN and preconditioned with 6 ml of H_2_O/ACN (5:95, v/v) (Fig. 2C). Then, 1000 μl of ACN, together with 500 μl of the sample after protein precipitation, were added onto the column. nLs including MGs, DGs, TGs, Chol Ester, and Cers were eluted with 4.5 ml of H_2_O/ACN (5:95, v/v). The second fraction, containing all choline-bearing PLs (i.e., lyso-PCs, PCs, and SMs), was collected with 5.5 ml of H_2_O/ACN (11:89, v/v). The third fraction, comprising lyso-PEs and PEs, was isolated using 10 ml of MeOH with 0.1% acetic acid. Subsequently, 18 ml of MeOH with 0.1% acetic acid was used to remove free oxylipins and FAs. PGs were eluted with 12 ml of H_2_O/ACN/IPA (6:18:76, v/v/v), and PIs and PSs were simultaneously isolated using 12 ml of H_2_O/ACN/IPA (20:35:45, v/v/v). Both solvents for PG and PI/PS elution contained 20 mM ammonium formate and 0.1% formic acid. Individual lipid fractions were eluted under a slight vacuum (∼900 mbar) and collected in borosilicate glass tubes containing 8 μl of 30% glycerol in MeOH. After collection, each fraction was spiked with deuterium-labeled oxylipin ISs (each 1 pmol/100 μl plasma/cell extract (∼400 μg cell protein)), and solvents were evaporated using a CHRIST CT 02–50 rotary evaporator (30°C, 1 mbar). The residues were reconstituted in 100 μl ACN/IPA (50:50, v/v). Lipid extracts were either analyzed by non-targeted LC-high-resolution-MS (LC-HRMS) (30) for method characterization described in the supplementary information (supplemental Fig. S5–S12) or prepared for the quantification of total oxylipins/FAs. For this, the reconstituted fractions were mixed with 300 μl IPA and centrifuged. Hydrolysis was carried out following the addition of 100 μl water and 100 μl 0.6 M KOH in H_2_O/MeOH 25:75, v/v) for 30 min at 60°C.

### Quantification of oxylipins and fatty acids

The analysis of free and total oxylipins was conducted as described elsewhere (23, 31, 32, 33, 34). Briefly, after protein precipitation in organic solvent, oxylipins were extracted either directly (free oxylipins, Fig. 2A), or after alkaline hydrolysis (23) (total oxylipins, Fig. 2B) using mixed-mode anion exchange Oasis Max cartridges, (3 ml, 60 mg sorbent per cartridge, particle size 30 μm, from Waters, Eschborn, Germany. LC-MS/MS oxylipin analysis was performed by external calibration with ISs using a 1290 Infinity II LC System (Agilent Technologies, Waldbronn, Germany) coupled to a QTRAP5500 AB Sciex mass spectrometer operating in scheduled selected reaction monitoring mode. FAs were quantified using the same sample preparation as described elsewhere (35). LC-MS/MS FA analysis was performed by external calibration with ISs using a 1260 LC System (Agilent Technologies, Waldbronn, Germany) coupled to an API 3200 instrument (AB Sciex, Darmstadt, Germany) operating in scheduled selected reaction monitoring mode. Detailed information regarding the oxylipin analysis including all information according to (36) are found in the SI. Moreover, information is reported in the Lipidomics Minimal Reporting Checklist (37) (https://doi.org/10.5281/zenodo.17881199).

### n3-PUFA supplementation study

The effect of n3-PUFA supplementation on the esterification of oxylipins in different lipid classes was investigated using human plasma samples derived from a double-blinded, randomized, controlled intervention trial (38). A subset of 9 participants (3 males, 6 females, age 23–72 years) was selected (supplemental Fig. S1). Four times a week, each participant received n3-PUFA capsules containing EPA and DHA (1.5 g and 1.8 g, respectively) from fish oil as re-esterified TG equivalent to 4 portions of fatty fish per week. Plasma samples of the subjects at baseline and after 12 months of n3-PUFA supplementation were analyzed.

## Results

Lipids were fractionated into nLs and four different PL classes (i.e., PC, PE, PG, and PI/PS), enabling the analysis of the binding form of oxylipins and their precursor PUFAs (Fig. 2). For this, a new SPE fractionation protocol using only a single cartridge was developed. The separation efficiency of each lipid class was evaluated using deuterium-labeled lipids (SPLASH) spiked to plasma and cell samples before lipid fractionation (Table 1). Based on the high recoveries, high separation efficiency and minimal cross-contamination (<5%) were achieved for most spiked deuterium-labeled lipid standards. In fraction 1 nLs were isolated, including TG 15:0/18:1[D7]/15:0 (99.6%), DG 15:0/18:1[D7]/0:0 (99.9%), MG 18:1[D7]/0:0/0:0 (99.9%), and Chol Ester 18:1[D7] (91%); no contamination with PLs was observed. Fraction 2 contained choline-bearing PLs, such as PC 15:0/18:1[D7] (99.8%), lyso-PC 18:1[D7]/0:0 (45%), and SM 18:1;O2/18:1[D9] (97%). Fraction 3 comprised PE 15:0/18:1[D7] (99.9%), lyso-PE 18:1[D7]/0:0 (99.6%), and 55% of lyso-PC 18:1[D7]/0:0. Free oxylipins and free FAs were quantitatively eluted from the cartridge with 18 ml MeOH + 0.1% acetic acid (supplemental Fig. S2). In fraction 4, PG 15:0/18:1[D7] (95%) was isolated. In fraction 5 both PS 15:0/18:1[D7] (99.9%) and PI 15:0/18:1[D7] (99.9%) were eluted. Of note, the elution of PG, PI, and PS required a minimum concentration of 10 mM ammonium formate (supplemental Fig. S3).

**Table 1:** Separation efficiency of the lipid class fractionation by SPE

Lipid Species	Fraction 1	Fraction 2	Fraction 3	Fraction 4	Fraction 5
Chol Ester 18:1[D7]	91 ± 2	4 ± 1	2.7 ± 0.4	1.6 ± 0.2	0.2 ± 0.1
TG 15:0/18:1[D7]/15:0	99.6 ± 0.2	0.2 ± 0.1	0.2 ± 0.1	0.1 ± 0.1	0.1 ± 0.1
DG 15:0/18:1[D7]/0:0	99.9 ± 0.1	0.1 ± 0.1	n.d.	n.d.	n.d.
MG 18:1[D7]/0:0/0:0	99.9 ± 0.1	n.d.	n.d.	n.d.	n.d.
PC 15:0/18:1[D7]	n.d.	99.8 ± 0.2	0.2 ± 0.2	n.d.	n.d.
SM 18:1;O2/18:1[D9]	n.d.	97 ± 2	2.7 ± 1.6	n.d.	n.d.
Lyso-PC 18:1[D7]/0:0	n.d.	45 ± 5	55 ± 5	n.d.	n.d.
PE 15:0/18:1[D7]	n.d.	0.1 ± 0. 1	99.9 ± 0.1	0.1 ± 0.1	n.d.
Lyso-PE 18:1[D7]/0:0	n.d.	n.d.	99.6 ± 0.1	0.4 ± 0.1	0.1 ± 0.1
PG 15:0/18:1[D7]	n.d.	0.3 ± 0.1	4 ± 1	95 ± 1	1.3 ± 0.1
PI 15:0/18:1[D7]	n.d.	n.d.	n.d.	n.d.	99.9 ± 0.1
PS 15:0/18:1[D7]	n.d.	n.d.	n.d.	n.d.	99.9 ± 0.1

A mixture of isotopically labeled lipid standards (SPLASH) was spiked to plasma and separated into five fractions. Shown are the relative recoveries of each standard in each fraction, reflecting the efficiency of lipid class separation across the fractions. Analysis was carried out using untargeted RP-LC-ESI-HRMS in Full MS/ddMS^2^ TOP N mode. Data is shown as mean ± SD (n = 3).

The robustness of the separation process was shown on three different days and for different biological samples, i.e., human plasma and HEK293 cell extract (supplemental Table S1). None of the tested matrices had a negative influence on the separation efficiency. When comparing the amount of recovered lipid standards added before fractionation (pre-spiked) and after the fractionation (post-spiked), more than 80% of each IS was recovered (supplemental Fig. S4).

**Fig. 3:**
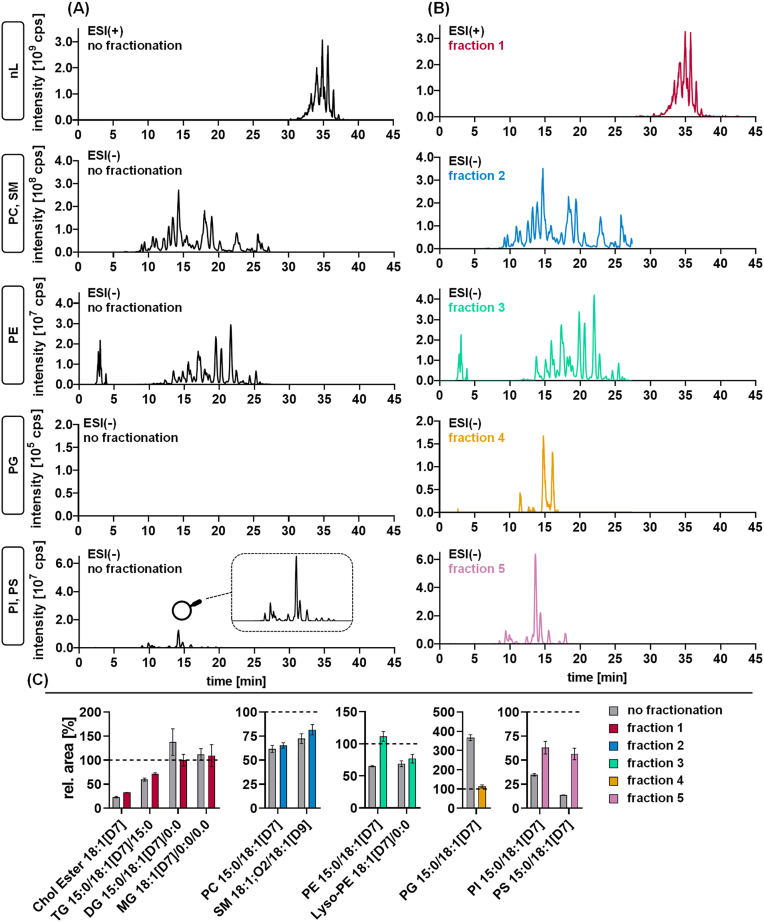
LC-HRMS lipidomics analysis of plasma with and without SPE-fractionation. Shown are cumulated XICs (sum of all detected species) of nLs and different PL classes in human plasma (A) without or (B) with fractionation. XICs were defined based on validated lipid features (lipid class level) using MS-DIAL (supplemental Table S3). C: Comparison of the recovery of ISs in the fractions to the non-fractionated sample showed a reduction of ion suppression effects for PE, PG, and PI/PS. ISs were spiked in plasma samples before extraction. Analysis was carried out using untargeted RP-LC-ESI-HRMS in Full MS/ddMS^2^ TOP N mode. Shown are the rel. IS areas (mean ± SD) of repeated extraction and analysis (n = 3) of a pool of human plasma.

To demonstrate that the developed SPE protocol also enables the separation of endogenous lipids in human plasma and HEK293 cells, MS-DIAL was used for data analysis following untargeted LC-HRMS analysis of intact lipids. Based on the detected features (supplemental Fig. S5 and supplemental Tables S2–S5), cumulative extracted ion chromatograms (XIC) were generated for each lipid class (TG, Chol Ester; PC, SM; lyso-PE, PE; PG; PS, PI). These were compared between the non-fractionated lipid extract (Fig. 3A) and fractionated lipid extracts of plasma and HEK293 cells (Fig. 3B and supplemental Fig. S6). The cumulative XICs (peaks, retention time, and abundance) were similar between non-fractionated samples and the respective lipid class fraction (Fig. 3A, B). This indicates that i) the lipids characterized in the non-fractionated samples are also present in the lipid class fraction and ii) endogenous lipids are efficiently separated into distinct fractions. Indeed, the peak pattern and the intensity of the XICs of fraction 1 (nLs) were identical to the non-fractionated plasma, supporting that all nLs were eluted in this fraction. Similarly, the chromatographic pattern of PC and SM species in the non-fractionated extract was recovered in fraction 2 (PC, SM), indicating a high recovery of the different choline-bearing PLs. In fraction 3 (PE), the cumulative XICs of PE features displayed a similar chromatographic pattern but with higher intensities compared to the non-fractionated sample. This is also reflected in a higher recovery of the IS PE 15:0/18:1[D7] (Fig. 3C): 110% in fraction 3 versus 65% in non-fractionated sample. For the earlier eluting IS lyso-PE 18:1[D7]/0:0, a comparable recovery was found between fractionated and non-fractionated plasma extracts. This suggests that compounds co-eluting with PE—presumably abundant PC and SM species—suppressed the PE signals in the non-fractionated sample. A similar and even more striking matrix effect was observed for PG. While PG species were not detected in the non-fractionated human plasma lipid extract (Fig. 3A), several PG species with a characteristic peak pattern were detected in fraction 4 (Fig. 3B). The lack of detection in the non-fractionated plasma extract was partially caused by a co-elution of almost isobaric (<3 ppm) M+1 ions of PE-O species (supplemental Fig. S7). Again, the matrix effects were observed in the recovery of the IS PG 15:0/18:1[D7]: strong “ion enhancement” in the non-fractionated extract (recovery 370%) versus no effects in fraction 4 (recovery 110%) (Fig. 3C, supplemental Fig. S8). Such “ion enhancement” effect of human plasma matrix on IS PG 15:0/18:1[D7] in RP-LC lipidomics has already been described (30). Fractionation also increased the abundance of PI/PS features in fraction 5 compared to the non-fractionated lipid extract. The recovery of the ISs PS 15:0/18:1[D7] and PI 15:0/18:1[D7] was also improved, indicating that PI/PS can be more sensitively detected following fractionation.

**Fig. 4:**
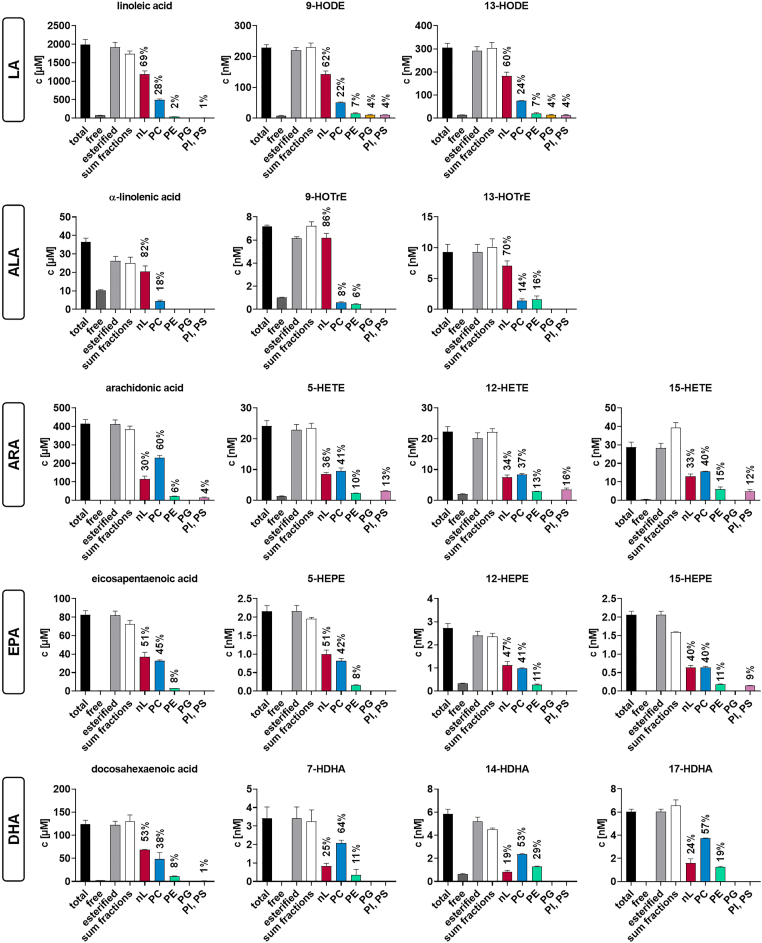
Lipid class-specific concentrations of esterified PUFAs and oxylipins in human plasma. Total FAs and oxylipins in the five lipid fractions of human plasma (pool of 3 healthy subjects) were quantified by targeted RP-LC-ESI(−)-MS/MS following alkaline hydrolysis (Fig. 2C). A representative set of PUFAs and hydroxy-PUFAs from LA, ALA, ARA, EPA and DHA is shown in each fraction and compared to the results without fractionation (total and free FAs/oxylipins, Fig. 2A, B). The concentrations of all detected oxylipins can be found in supplemental Table S8. Shown are the concentrations (mean ± SD) of repeated extraction and analysis (n = 3) of a pool of human plasma.

The SPE fractionation protocol was developed to quantitatively determine the distribution of oxylipins and PUFAs in lipid classes. For this purpose, oxylipins and FAs were released from each lipid fraction by an established alkaline hydrolysis procedure (22, 23, 33), and the concentrations of a comprehensive set of hydroxy-PUFAs were quantified in two pools of human plasma (supplemental Tables S8 and S9). Figure 4 shows the concentrations of a representative set of hydroxy-PUFAs derived from LA, ALA, ARA, EPA, and DHA, along with their precursor PUFAs. The concentrations of total and free FAs and oxylipins were comparable to previous reports from human plasma (10, 24, 39, 40). LA-derived hydroxy-PUFA (HODEs) showed a manyfold higher concentration than all other oxylipins. In agreement with previous studies (14, 22, 41, 42, 43), the vast majority (≥85%) of hydroxy-PUFAs was detected in esterified form. The sum of FAs and oxylipins recovered from the SPE fractions closely matched the concentrations obtained in the non-fractionated human plasma samples. This indicates a high recovery of esterified FAs/oxylipins and low loss or artificial formation/degradation during the fractionation procedure, enabling the evaluation of the distribution of the esterified oxylipins in the different lipid classes: Hydroxy-LAs and -ALAs (HOTrEs), and their precursors were predominantly esterified to nLs (60%–86%). Hydroxy-ARAs (HETEs) and ARA were more prominently bound to PLs (66%–70%). EPA, hydroxy-EPAs (HEPEs) and DHA were approximately equally distributed between nLs and PLs while hydroxy-DHAs (HDHAs) occurred predominantly in PLs (75%–86%).

Among PL classes, LA, ALA, and their oxylipins were predominantly found in fraction 2, which included both PC and SM. Because SM species are composed of unsaturated or monounsaturated fatty acyl chains (44, 45), we conclude that these (hydroxy-)PUFAs are primarily bound to PC. In contrast, minor amounts were bound in PE, and only negligible amounts were found in PG and PI/PS. HETEs were predominantly esterified to PC (37%–41%), followed by PE (10%–15%), and PI/PS (12%–16%). HEPEs showed comparable concentrations in nLs (40%–51%) and in PC (40%–42%) and were less esterified to PE (8%–11%). Notably, 15-HEPE showed a relative concentration of 9% in PI/PS. The relatively high esterification to PI/PS was unique to HETEs and 15-HEPE. HDHAs, such as 7- and 14-HDHA were dominantly found in PC (53%–64%) and PE (11%–29%) but not detected in PG and PI/PS. Almost the same distribution of oxylipins was found in plasma pool 2 (NIST plasma, SRM 1950) (supplemental Table S9).

**Fig. 5:**
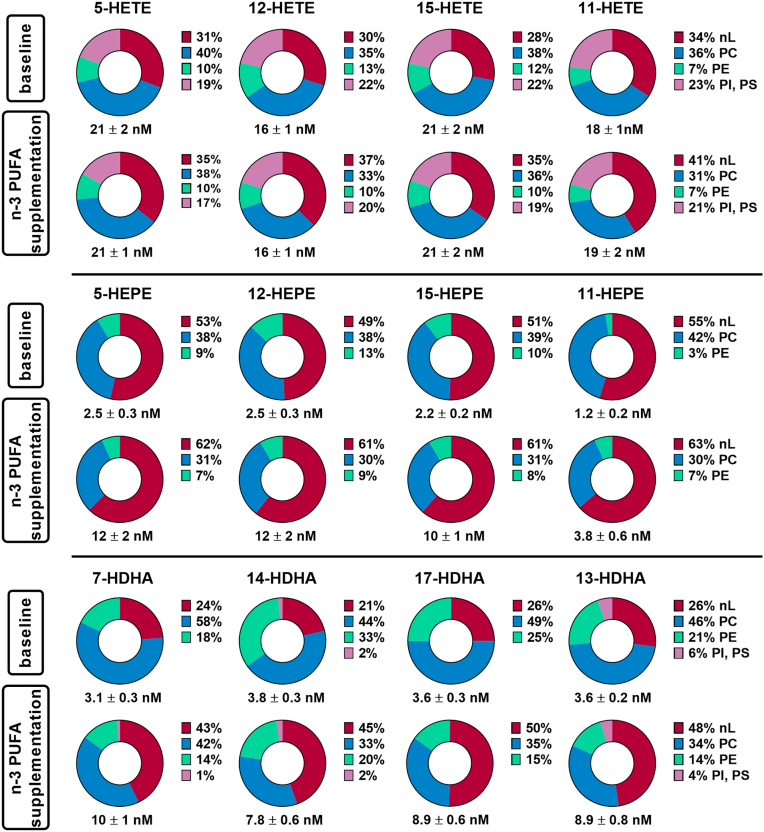
Change in the concentration and distribution pattern of esterified hydroxy-PUFAs across lipid classes in human plasma following 12 months of n3-PUFA supplementation. Total oxylipins were quantified in the lipid class fractions in plasma of human subjects at baseline and after 12 months of n3-PUFA supplementation (1.5 g EPA and 1.8 g DHA per portion; 4 portions per week). Shown is the concentration of total oxylipins and their relative distribution in the lipid classes of a representative set of hydroxy-PUFAs. The concentration of oxylipins in each lipid fraction can be found in supplemental Table S10. Analysis was carried out using targeted RP-LC-ESI(−)-MS/MS following alkaline hydrolysis. Shown are the mean values ± SEM (n = 9).

Next, we investigated in which lipid classes the well-described changes (14, 46, 47) in esterified oxylipins occur following 12 months of n3-PUFA supplementation (Fig. 5). EPA and DHA supplementation did not change the concentration or distribution patterns of HODEs, HOTrEs, HETEs, and their n6-PUFA precursors (supplemental Table S10). In contrast, n3-PUFA supplementation led to a marked increase in several HEPEs and HDHAs within the different lipid classes and shifted their distribution into lipids toward nLs. 5-HEPE and 12-HEPE increased from 2.5 ± 0.3 nM to 12 ± 2 nM, 15-HEPE from 2.2 ± 0.2 nM to 10 ± 1 nM, and 11-HEPE from 1.2 ± 0.2 nM to 3.8 ± 0.6 nM. The proportion of 5-HEPE in nLs was elevated from 53% to 62%, of 12-HEPE from 49% to 61%, of 15-HEPE from 51% to 61%, and of 11-HEPE from 55% to 63% after n3-PUFA supplementation. Following supplementation, 7-HDHA, 14-HDHA, 17-HDHA, and 13-HDHA showed concentrations in the same range as HEPEs (Fig. 5), despite higher baseline levels. 7-HDHA increased from 3.1 ± 0.3 nM to 10 ± 1 nM, 14-HDHA from 3.8 ± 0.3 nM to 7.8 ± 0.6 nM, 17-HDHA from 3.6 ± 0.3 nM to 8.9 ± 0.6 nM, and 13-HDHA from 3.6 ± 0.2 nM to 8.9 ± 0.8 nM. Again, this increase occurred largely in the nL fraction: 24%–43% for 7-HDHA, 21%–45% for 14-HDHA, 26%–50% for 17-HDHA, and 26%–48% for 13-HDHA. Overall, supplementation of n3-PUFA resulted in elevated concentrations of EPA and DHA-derived hydroxy-PUFAs in nLs, PC and PE classes, with the strongest increase in nLs (∼600%) (Fig. 5., supplemental Fig. S17). Interestingly, the supplementation also led to a slight increase in ARA-derived hydroxy-PUFA in nLs (supplemental Fig. S17, supplemental Tables S10, and S11). The relative amount of hydroxy-PUFA to their precursor PUFA in each lipid class was determined (supplemental Fig. S18 and supplemental Table S12). At baseline and following n3-PUFA supplementation, the rates were similar in ranges between 0.01% and 0.15%. The highest ratio was found for ARA in fraction 5 (PI, PS).

This lipid class-specific analysis of oxylipins in plasma shows that hydroxy-PUFAs are precursor-dependent esterified to distinct lipid classes and that their lipid class distribution can be shifted with the diet.

## Discussion

A novel lipid class SPE-fractionation method was developed using silica-bonded aminopropyl material as the solid phase. This material has been widely applied in previous lipid fractionation approaches (25, 26, 48). In contrast to commonly used normal-phase LC (NP-LC), our method employed a HILIC-based approach, which has not yet been reported in this context. This approach reduces the use of environmentally hazardous chemicals such as halogenated solvents typically employed in NP-LC. Only a single column is needed to separate the lipids into the classes nLs; PC, SM; PE; non-esterified FA and oxylipins; PG; and PI, PS with high efficiency. This was shown for the recovery of the spiked labeled lipids in human plasma as well as HEK293 cell extracts. Endogenously present lipids in the samples were also efficiently separated as demonstrated based on lipid class-specific XICs created using MS-DIAL (Fig. 3). The chromatographic peak patterns and signal intensities of isolated lipid classes with and without fractionation were similar. Of note, the isolation of the lipid classes led to a strong reduction of ion suppression, improving the LC-MS detection. This could be helpful, particularly for the analysis of low-abundant PL classes by untargeted lipidomics.

Compared to SPE-based lipid fractionation protocols previously described, the performance of the separation reported here is better than those using a single column, as they only separated nLs and PLs (49, 50) or did not separate PL classes as effectively, e.g., only isolating PC (51, 52). Methods that have a comparable separation efficiency or which can separate five or more lipid fractions employ two or more different SPE cartridges (25, 26, 27). Three cartridges were used to separate nLs into MGs, DGs, TGs, and CEs (26, 27) or to separate PLs into PG; PA, PI, PS; PE; as well as lyso-PC, PC and SM (25). Here, we achieved comparable separation efficiencies of the PL classes using only one column, which is ideal for our aim to pinpoint the binding form of esterified oxylipins in major lipid classes. The time needed for sample preparation is thereby short, reducing the risk of artificial formation and degradation, which is a challenge in oxylipin analysis (36). However, low cross-contaminations between lipid classes, as observed for Chol Ester 18:1[D7] eluting with 2%–4% in the other fractions, must be taken into account when results are interpreted.

Quantification of esterified oxylipins in the different lipid fractions after hydrolysis offers better sensitivity compared to direct analysis of intact lipids. Lipid fractionation enabled the detection of hydroxy-PUFAs in PE, PG, and PI/PS in human plasma (Fig. 4), which was not reported using a non-targeted (semi-quantitative) LC-HRMS/(MS) analysis (16). Also, with a more sensitive targeted LC-MS/MS method on a triple quadrupole instrument, the detection of hydroxy-PUFAs in PLs was not possible in HEK293 cells at baseline (19). This may be explained by the distribution of the oxylipins across a large number of lipid classes and species (Fig. 1). For example, the detection of six hydroxy-PUFAs in the targeted LC-MS/MS oxPL method was based on 63 oxPLs with 190 transitions (three for each oxPL species). The HILIC-based fractionation approach is less specific than direct analyses but provides better sensitivity and covers more oxylipins. Only one chromatographic peak per oxylipin in each lipid fraction results, allowing a sensitive detection. Moreover, the use of authentic oxylipin standards together with a validated LC-MS/MS method (23, 31, 32, 33, 34) enables reliable quantification.

The developed fractionation approach was applied to analyze two independent human plasma pools, including NIST SRM 1950 (supplemental Table S9). The esterified oxylipins present in the samples were quantitatively detected following fractionation because the sum of the concentrations in the lipid fractions matched the concentration of esterified oxylipins determined without fractionation (>80%). Owing to that, this approach allows to quantitatively determine the amount of oxylipins bound in nLs and different PL classes in human plasma. Earlier SPE-based studies only enabled the differentiation of esterified oxylipins between nLs and PLs (29, 53). Watanabe *et al.* investigated the distribution of endogenous epoxy-PUFAs in rat plasma and found that 37% of 11(12)-EpETrE was bound in nLs and 67% in PLs, which is comparable to the distribution of HETEs in human plasma (Fig. 4) (29).

Regarding the binding form of hydroxy-PUFAs in plasma, we found striking precursor-dependent differences (Fig. 4). Oxylipins derived from LA and ALA were preferably bound in nLs. This is consistent with studies investigating oxidized lipids directly. Here, the majority of oxidized LA and ALA was found in nLs (16).

In the Western Diet, which includes high amounts of LA-rich vegetable oils, the consumption of LA (up to 29 g/day) (54) and ALA (up to 2.3 g/day) (55) is much higher compared to the intake of ARA (up to 0.35 g/day) (56) and DHA (up to 0.50 g/day) (57). With that, also considerable amounts of oxylipins are ingested (58, 59, 60), which are bound to TGs. In plasma, the concentrations of LA-derived oxylipins were many-fold higher compared to oxylipins derived from other PUFAs, consistent with the high consumption of LA and ALA. Because we also found LA and ALA-derived oxylipins in nLs, we assume that these plasma oxylipins may be derived from direct uptake from the diet or from (aut)oxidation of the ingested TGs.

Oxylipins derived from long-chain PUFAs such as ARA and DHA were predominantly esterified to PLs. ARA and DHA are the main PUFAs occurring in cell membranes and are critical for membrane fluidity as well as cell function (61). Moreover, both serve as precursors of bioactive lipid mediators (62, 63). That they, as well as EPA, were found esterified to PLs in plasma suggests that they were released from cells, e.g. as vesicles or in lipoproteins.

All analyzed oxylipins showed a distinct distribution among the PL classes. PC is the major PL class in human plasma and contains a large proportion of plasma total FAs (∼40%) (45, 64). Thus, it is not surprising that PC is the dominating PL class in which oxylipins are bound in plasma and that all the investigated hydroxy-PUFAs occur at least in part in PC (Fig. 4). Nonetheless, precursor-dependent differences were found: ARA-derived oxylipins and 15-HEPE were present at relevant concentrations in the PI/PS fraction. In cells, ARA is not randomly distributed among PL classes but is preferentially incorporated into PC and PI (65, 66). Here, ARA-PIs are one of the precursors for inositol phosphate signaling pathways (67). The occurrence of ARA, its oxylipins and 15-HEPE in PI in plasma may reflect the export of PI from cells, e.g. in the form of vesicles, membrane fragments, or during lipoprotein formation in the liver. While Maskrey *et al.* reported preferential incorporation of 15-HETE into PE in activated monocytes (20) suggesting a direct oxidation of PE by 15-LOX-1, we recently showed that in HEK293 cells, 15-HETE and also 15-HEPE were almost exclusively incorporated into PI via the Lands’ cycle (13, 19). This is consistent with earlier studies using isotopically labeled oxylipins (68, 69). Unlike cell membranes, plasma lipids reflect in addition systemic processes such as lipoprotein metabolism, vesicle transport, or liver-associated export of lipids (70). This may explain why also other ARA-derived oxylipins are found in PI, such as 5-HETE and 12-HETE, which were esterified to PC (and not PI) in HEK293 cells (19).

In plasma, DHA and its derived oxylipins showed the highest relative esterification to PE compared to all other lipids. DHA is known to be preferentially enriched in PE in the inner leaflet of membranes, e.g. as a regulator of membrane fluidity in human cells (71). The release of oxPE would thus explain the high concentration of DHA and its derived oxylipins in the PE fraction. Of note, consistent with our studies in HEK293 cells (13, 19), DHA-derived hydroxy-PUFAs do not occur in PI in human plasma.

We then asked the question whether n3-PUFA supplementation changes the oxylipin binding forms in human plasma. It is well described that the composition of non-esterified (72, 73, 74, 75, 76) and esterified oxylipins (14, 46, 77, 78) in plasma is dramatically changed by n3-PUFA supplementation. At baseline, the participants showed the same distribution of HETEs, HEPEs, and HDHAs over the lipid classes as the two other analyzed plasma pools (supplemental Figs. S13–S15). Consistent with earlier studies (75, 76), n3-PUFA supplementation hardly changed the concentration of HETEs, while HEPEs and HDHAs increased dramatically (Fig. 5). EPA showed a greater relative increase due to its lower baseline level (46, 78) (supplemental Fig. S16). This increase in plasma concentrations of n3-PUFA-derived oxylipins by supplementation can be explained in different ways. First, the oxylipins are present in the (fish oil) n3-PUFA supplements due to autoxidation (79), which are then absorbed together with n3-PUFAs and released in the bloodstream. Ostermann *et al.* reported a perfect dose-dependent increase of n3-PUFA-derived non-esterified oxylipins following n3-PUFA supplementation in plasma (46), which could be well explained by the co-ingestion and absorption of HEPEs and HDHAs with their precursor PUFAs, EPA and DHA, respectively. The second explanation is that the n3-PUFA are first absorbed, integrated into lipids, and the oxylipins are then formed by autoxidation or enzymatically, e.g., by LOX (6). Oxidation of PUFAs can occur with or without release by lipase and re-esterification by Lands’ cycle enzymes (80). Because LOX products such as 15-HEPE or 17-HDHA are similarly elevated as autoxidatively formed 11-HEPE and 13-HDHA, enzymatic formation may not be the dominating factor. Consistently, the supplementation did not change the ratio of hydroxy-PUFA to the precursor PUFA (supplemental Fig. S18).

Although supplementation increased the concentration of HEPEs and HDHAs in all lipid classes, the highest elevation was surprisingly found in nLs. This distinct increase supports a direct absorption of oxylipins alongside the n3-PUFAs, similar to that discussed for LA and ALA. The biological roles of esterified oxylipins in plasma remain poorly understood. Shearer and Newman suggest that esterification serves as a mechanism to regulate the biological activity of oxylipins (81). Based on that, the elevated intake/formation of oxylipins following supplementation could thus result in an increased esterification into nLs to control the bioactivity of the oxylipins functioning as a reservoir. The esterified oxylipins can be released from lipoproteins such as VLDL by lipoprotein lipases and then may act on endothelial cells (72, 81, 82). Our data shows that even after 12-month supplementation, the DHA-derived hydroxy-PUFAs occur in plasma more in nLs compared to baseline, suggesting a direct effect of the diet on the plasma oxylipins. Many studies aim to correlate plasma oxylipin levels with health status, ideally serving as biomarkers for diseases (83, 84, 85). The high variability in plasma oxylipins observed in these studies may be explained by direct effects from the diet, which warrant further investigation.

## Conclusion

This study provides novel insights into the lipid class-specific occurrence of oxylipins in human plasma. A new HILIC-based fractionation method enabled the efficient separation of lipids into nLs and four PL fractions using a single SPE cartridge. This approach allows the quantification of a large number of esterified oxylipins in lipid fractions and can readily be integrated with existing oxylipin analysis workflows. The data demonstrate in two independent pools of human plasma that esterification patterns of oxylipins in plasma are largely determined by their precursor PUFAs. Oxylipins derived from LA and ALA are largely bound to nLs, presumably reflecting the circulation of oxylipins which have been ingested with the diet alongside their precursor PUFAs. In contrast, oxylipins derived from the long-chain PUFAs ARA, EPA and DHA circulate in plasma primarily esterified in PLs, presumably reflecting their release from cellular membranes, e.g. as lipoproteins or vesicles. Moreover, the preferred esterification of ARA, its oxylipins and 15-HEPE to PI and of DHA and its oxylipins to PE in human cells is reflected in plasma lipids. Notably, n3-PUFA supplementation resulted in an overall increase of HEPEs and HDHAs combined with a significant redistribution of HDHAs toward binding in nLs. This raises new questions regarding the direct effect of the diet on the occurrence of esterified oxylipins in human plasma.

## Data availability

All data are contained within the article or supplementary material.

### Supplemental data

This article contains Supplemental data (13, 23, 24, 30, 32, 33, 34, 38, 49, 86, 87, 88).

### Ethical approval

Blood for the n3-PUFA supplementation study was collected with informed consent from healthy human subjects in accordance with the guidelines of the Declaration of Helsinki. The n3-PUFA supplementation study was approved by the Suffolk Local Research Ethics Committee.

## Conflict of interest

The authors declare that they do not have any conflicts of interest with the content of this article.

## Supplementary data

Supporting Tables

Oxylipin Method Report
