# Supplementary material for: Quantification of esterified oxylipins following HILIC-fractionation of lipid classes
Source: J Lipid Res. 2025 Nov 21;67(1):100950. doi: 10.1016/j.jlr.2025.100950 (PMC12799955; doi:10.1016/j.jlr.2025.100950)
Supplement: Supplemental information [file mmc1.pdf]

Quantification of esterified oxylipins following HILIC-fractionation of lipid classes

Supplemental data

Luca M. Wende[1], Laura Carpanedo[1], Lilli Scholz[1], Nadja Kampschulte[1], Annette L. West[2], Philip C. Calder[2,3], Nils Helge Schebb[1*]

[1] Food Chemistry, School of Mathematics and Natural Sciences, University of Wuppertal, 42119 Wuppertal, Germany,

[2] School of Human Development and Health, Faculty of Medicine, University of Southampton, Southampton SO16 6YD, UK

[3] NIHR Southampton Biomedical Research Centre, University Hospital Southampton NHS Foundation Trust and University of Southampton, Southampton SO16 6YD, UK

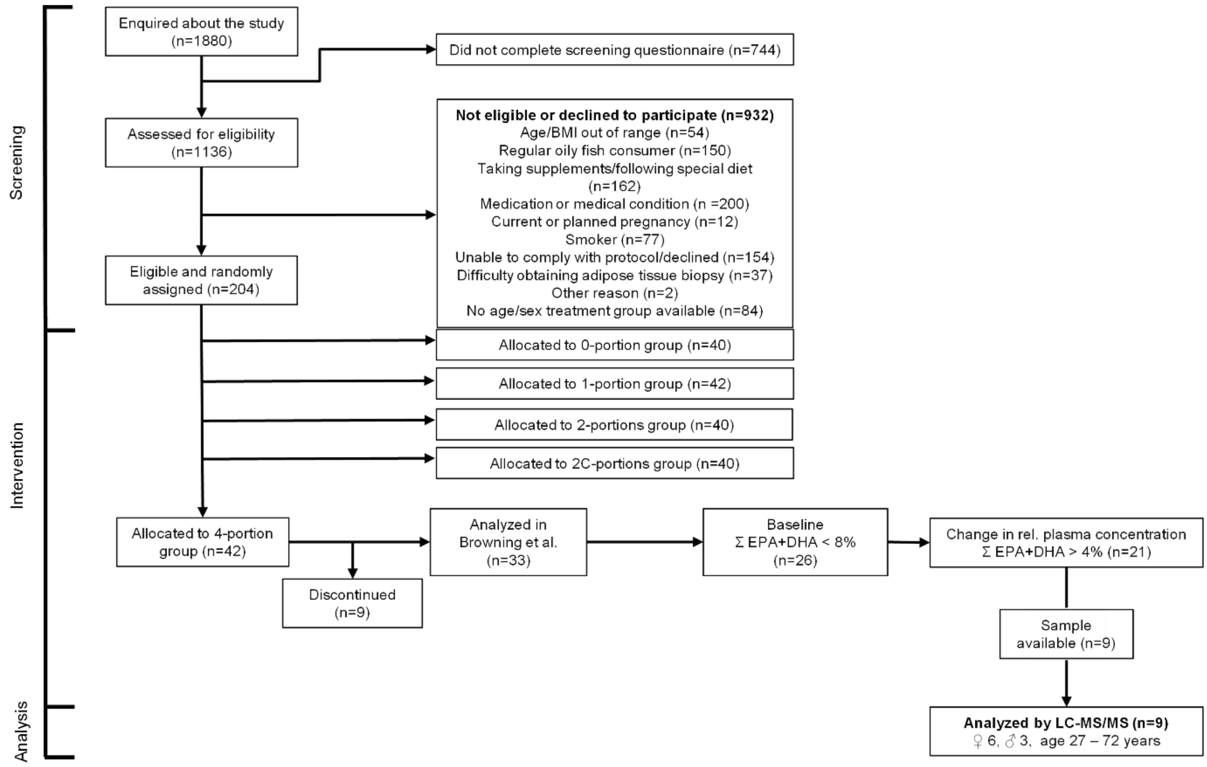

Fig S1: Design of the n3-PUFA supplementation study and sample selection

A subset of human plasma samples from an intervention study (1, 2) was used to investigate the pattern of esterified oxylipins in different lipid classes following n3-PUFA supplementation. On 4 days per week subjects received n3-PUFA capsules containing 1.5 g EPA and 1.8 g DHA per portion, corresponding to 4 portions of fatty fish a week (4-portion group). 9 participants (3 males, 6 females, age 23 – 72 years) out of 42 subjects were selected fulfilling the following criteria: Plasma samples had a relative level of EPA + DHA <8% of total FA at baseline and a change in the relative level of EPA + DHA >4% after 12 months of n3-PUFA supplementation (1). Available aliquots of the samples at baseline and after 12 months were properly stored at -80°C. Plasma samples were fractionated and esterified FA and oxylipins were analyzed after alkaline hydrolysis by LC-ESI(-)-MS/MS (3-5).

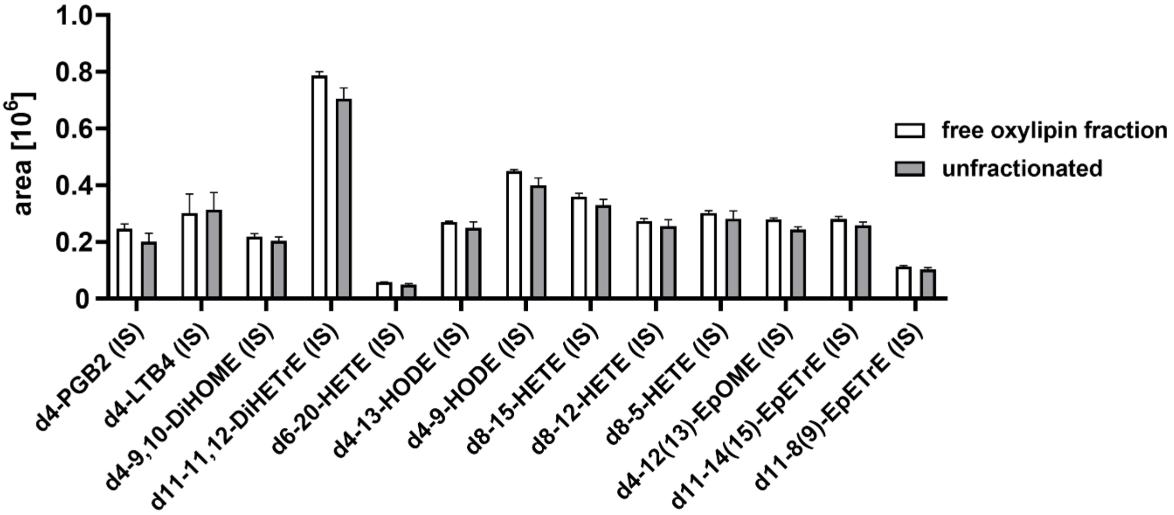

Fig. S2: Recovery of free oxylipins following SPE fractionation of lipid classes. Human plasma (100 µL) was spiked with a mixture of deuterium-labeled oxylipins followed by fractionation of lipid classes using SPE. The fraction containing free oxylipins was collected and free oxylipins were analyzed. The peak areas of the standards in the fraction were compared to those of the standard mixture directly analyzed without fractionation. The comparison of the peak areas shows a high recovery of free oxylipins after SPE indicating that free oxylipins are completely eluted by 18 mL MeOH with 0.1% acetic acid (Fig. 2) Analysis of oxylipins was carried out using targeted LC-ESI(-)MS/MS (3-5). Results are shown as mean ± SD (n = 3).

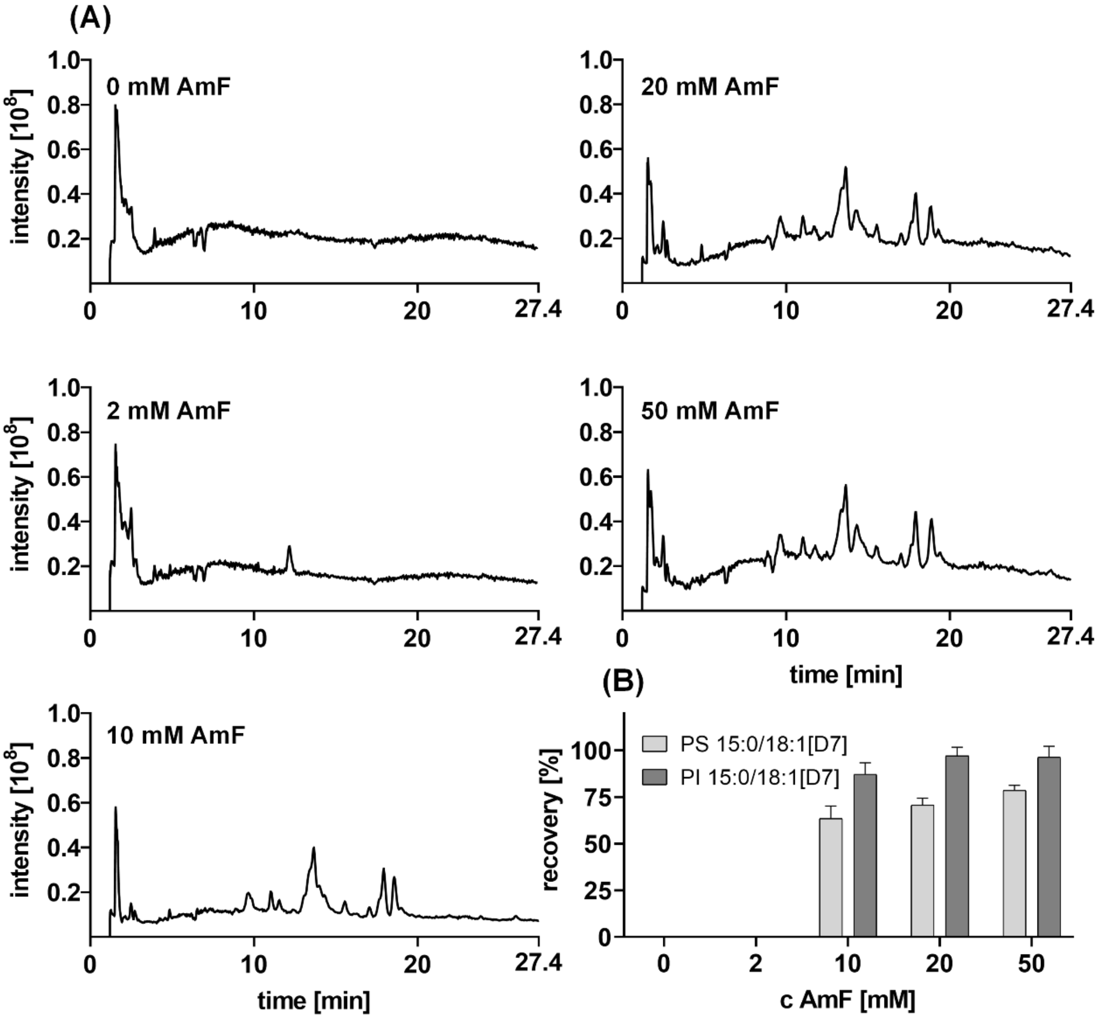

Fig. S3: Ammonium formate-dependent elution of PI and PS species in lipid fraction 5 **(A)** Shown are total ion chromatograms (*m/z* 200 - 1200) of SPE lipid fraction 5 of a HEK293 cell extract eluted separately with increasing concentrations of ammonium formate (AmF). The eluent was composed of H_2O/ACN/IPA (20:35:45, v/v/v) containing 0.1% formic acid and ammonium formate (0 – 50 mM). **(B)** Recovery of PI 15:0/18:1[D7] and PS 15:0/18:1[D7] with increasing ammonium formate concentration used for elution (n = 3). The results indicate that a minimum ammonium formate concentration of 10 mM in the eluent is required for effective elution of PI and PS species slightly improving with increasing ammonium formate concentration. However, 20 mM was chosen as higher concentrations lowered the efficiency of the following alkaline hydrolysis used to quantify esterified oxylipins in the fractions. Analysis was carried out using untargeted LC-ESI(-)-HRMS (Q Exactive HF) in Full MS/ddMS^2 TOP N mode (6).

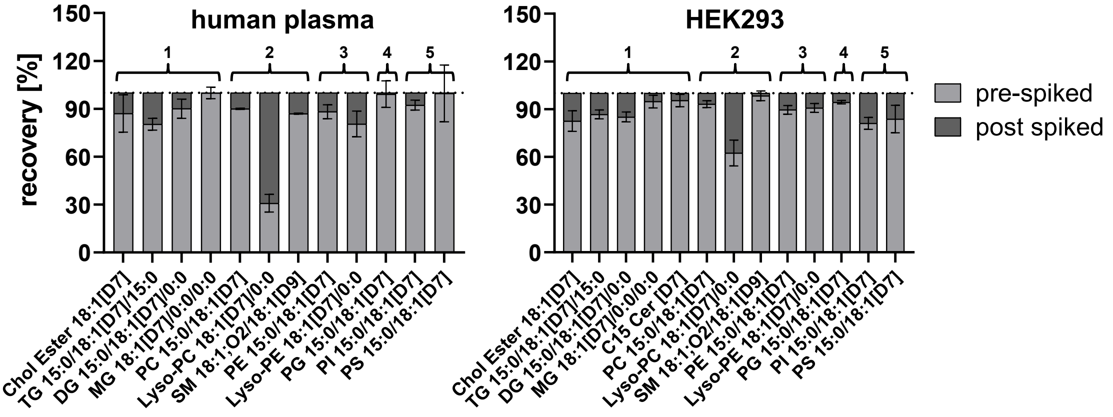

Fig. S4: Recovery of deuterium-labeled lipids (SPLASH standards) after lipid class fractionation using SPE

Human plasma (100 µL) or HEK293 cell homogenate (~400 µg protein) were extracted and spiked with SPLASH Lipidomix or EquiSPLASH, and samples were fractionated using SPE. Another set of both samples was spiked with the standards after SPE-fractionation in each individual fraction (post-spiked), which was set to 100% recovery. The results show high extraction recoveries of most standards after fractionation, with more than 80% recovered in each fraction. Only lyso-PC 18:1[D7]/0:0 shows lower recovery in fraction 2 because it partially coelutes in fraction 3. The number of the lipid fractions is shown at the top of the figure. Analysis was carried out using untargeted LC-ESI-HRMS (Q Exactive HF) in Full MS/ddMS^2 TOP N mode (6). Results are shown as mean ± SD (n = 3).

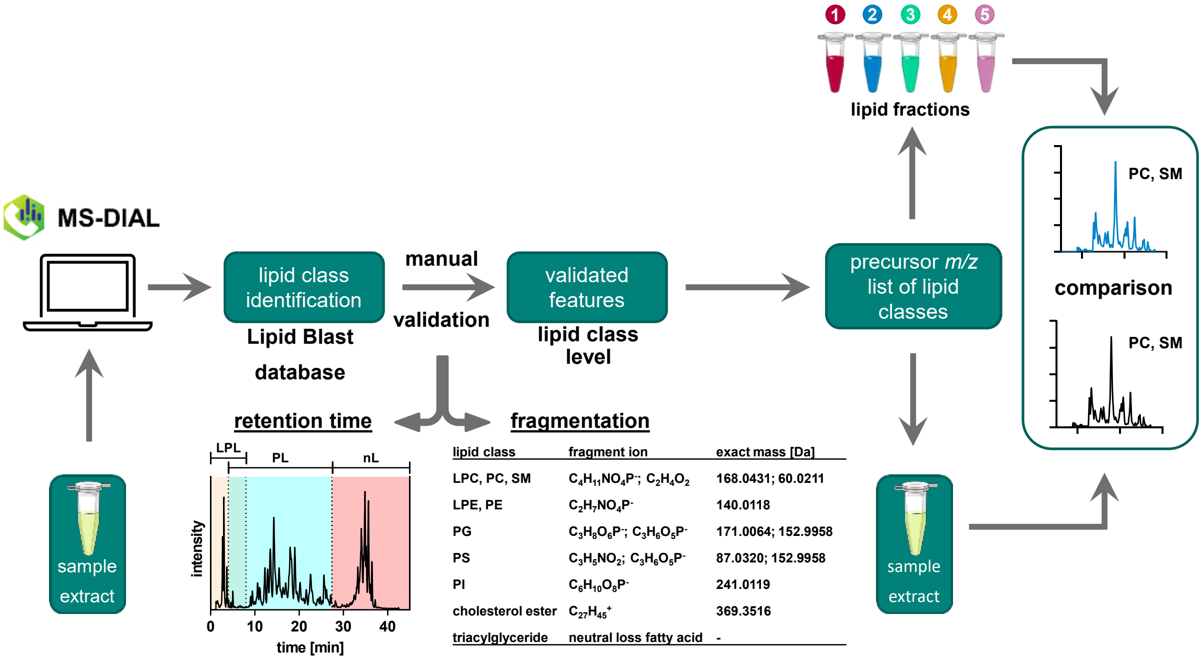

Fig. S5: Data processing for chromatographic lipid class pattern comparison Lipids were extracted and analyzed by untargeted LC-HRMS (Q Exactive HF Orbitrap) in Full MS/ddMS² TOP N mode (Table S7). Data evaluation was carried out in both ionization modes using MS-DIAL software (version 5.3) (7). The processing steps included feature detection, spectra deconvolution, and peak alignment across samples. Detailed data processing and parameter settings for MS-DIAL are listed in Table S6. For evaluation, only features matching the used spectral library were used (reference matched) (Tables S2 and S4). The features were manually validated for lipid class identification according to Liebisch *et al.* (8) based on plausible retention times and fragmentation criteria (Table S3 and Table S5). The retention time criteria for validating the features were as follows: lyso-PL (≤8 min), PL (≥4 min), SM (≥4 min), CE, and TG (≥27.4 min). Features outside these criteria were excluded from further evaluation. As fragmentation criteria, characteristic product ions of the lipid headgroup were used, such as *m/z* 168.0426 for PC and SM, *m/z* 140.0118 for PE, or *m/z* 241.0113 for PI in negative mode according to Pi *et al.* (9) (Fig. S9-S12). Validated features (lipid class level) were listed and sorted according to the SPE-based lipid class fractionation (Tables S2, S3). Theoretical precursor *m/z* values assigned to lipids eluting in the corresponding fraction were used to generate summed XICs for all lipids of the eluting lipid classes. XICs were generated for both non-fractionated samples and fractionated samples to assess lipid recovery within specific lipid classes after fractionation. Analysis was carried out using untargeted LC-ESI-HRMS (Q Exactive HF) in Full MS/ddMS² TOP N mode (6) with slight modifications. The following gradient was used: 0–0.7 min 30% B; 0.7–0.8 min 30–52.5% B; 1.5–11 min 52.5% B; 11–20 min 52.5–60% B; 20–40 min 60–99% B; 40–42 min 99% B; 42–45 min 30% B with a flow rate of 260 µL/min. The method included a polarity switch to detect different lipids in both negative and positive ionization modes within a single run. The analysis started in ESI(-) mode to detect and characterize polar lipids, such as PLs, and was switched to ESI(+) mode at 27.4 min to detect nL, such as TGs and CEs. Detailed instrument parameters are given in Table S7.

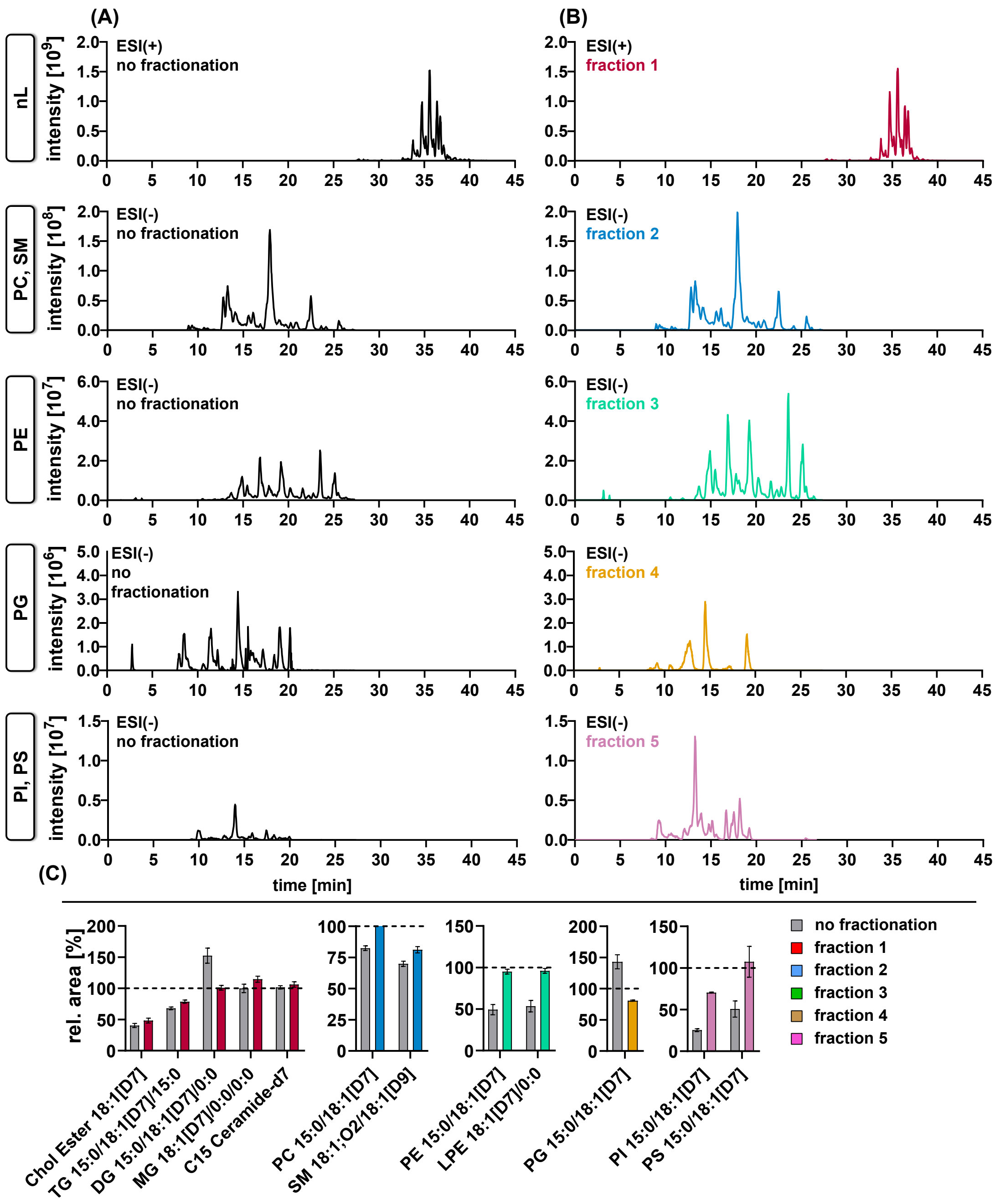

Fig. S6: SPE fractionation of lipid classes in HEK293 cells Shown are cumulated XICs (sum of all detected species, Fig. S5) of nLs and different PL classes in HEK293 cells **(A)** without and **(B)** with fractionation. XICs were defined based on validated lipid features using MS-DIAL (see Table S5). Theoretical precursor *m/z* of identified and validated features on lipid class level were used to build XICs in the non-fractionated and the fractionated samples according to the separated lipid classes in each fraction. **(C)** Recovery of IS in the fractions compared to the non-fractionated sample shows a reduction of ion suppression effects for PE, PG, and PI/PS.

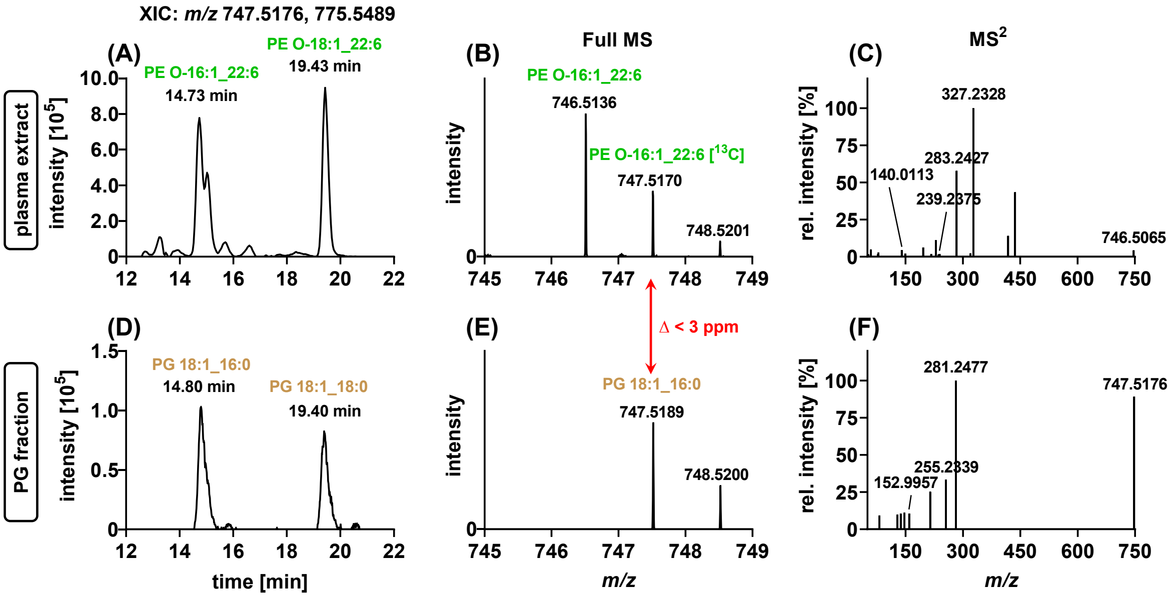

Fig. S7: Co-elution of PG and PE-O makes the detection of PG in biological samples by RP-LC-HRMS impossible, which can be solved by lipid class fractionation. **(A)** XIC of *m/z* 747.5182 and *m/z* 775.5443 (≤5 ppm) corresponding to the monoisotopic *m/z* of characterized PE O-16:1_22:6 and PE O-18:1_22:6 in a non-fractionated human plasma extract. **(B)** Zoomed Full MS spectrum at 14.73 min and **(C)** ddMS^2 scan of the precursor *m/z* 746.5136 (isolation window *m/z* 1.5). **(D)** XIC of *m/z* 747.5176 and *m/z* 775.5489 (≤5 ppm) corresponding to characterized PG species PG 18:1_16:0 and PG 18:1_18:0 in SPE fraction 4. **(E)** Zoomed Full MS and **(F)** ddMS^2 scan of *m/z* 747.5189 (isolation window ± *m/z* 1.5). **(A)** shows that co-eluting and higher abundant PE O-species overlay PG species in human plasma. The ions of the M+1 isotope of PE O-16:1_22:6 and PE O-18:1_18:0 are nearly isobaric (≤ 5 ppm), respectively, with PG 18:1_16:0 and PG 18:1_18:0 making it impossible (for MS-DIAL) to detect and characterize the PG species in the non-fractionated human plasma extract. The results show that fractionation can provide increased confidence in determining the lipid class of a lipid, as well as facilitating the detection and characterization of less abundant lipids that coelute with abundant isobaric lipids. Analysis was carried out using untargeted LC-ESI-HRMS (Q Exactive HF) in Full MS/ddMS^2 TOP N mode (6) with modifications described in Fig. S5.

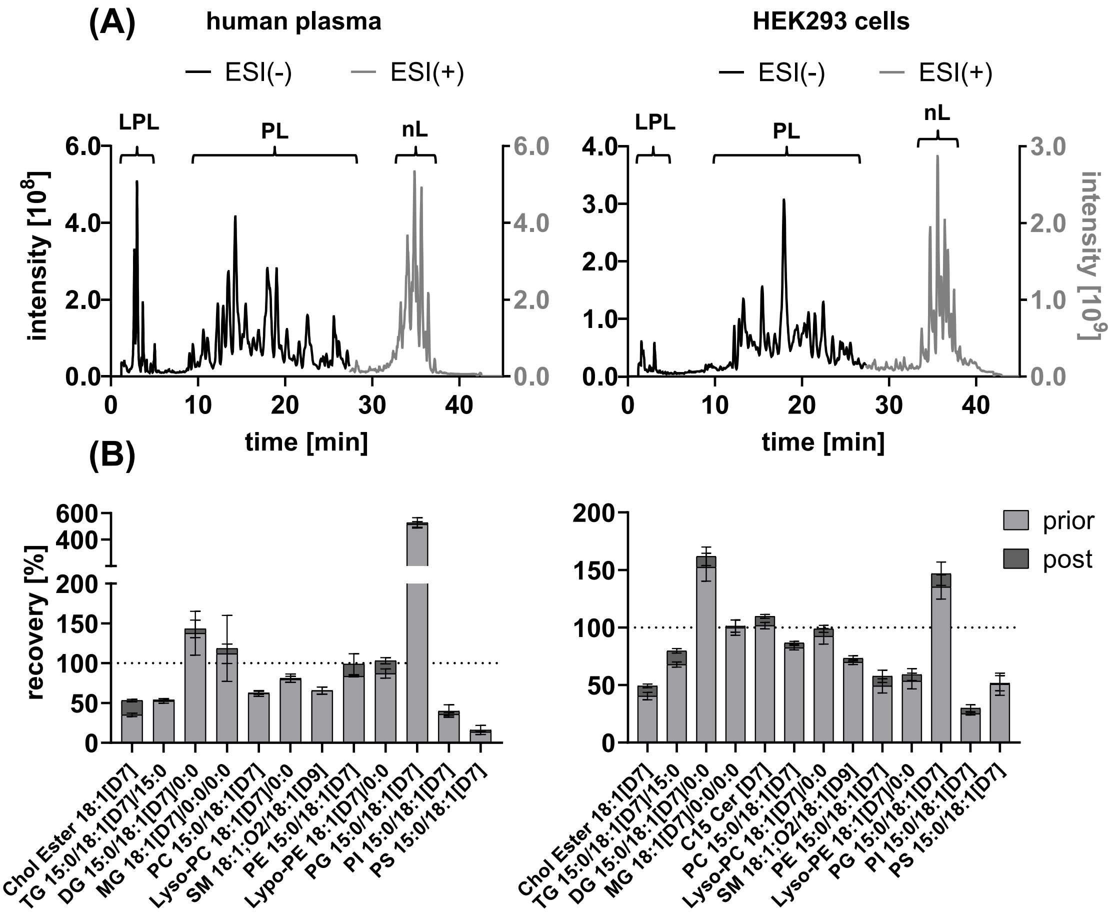

Fig S8: Analysis of human plasma and HEK293 cells using untargeted LC-HRMS (A) Total ion chromatograms of extracts from human plasma and HEK293 cells. (B) Recovery of ISs (SPLASH Lipidomics for human plasma and EquiSPLASH for HEK293T cells) after extraction. Samples were spiked with IS both either before (prior) or after (post) extraction, and IS peak areas were compared. The results indicate high extraction recovery (>80%) for most lipids. Severe matrix effects were observed, particularly for PI 15:0/18:1[D7] and PS 15:0/18:1[D7] in both, human plasma and HEK293 cells. Figures 3 and S6 demonstrate that these suppression effects can be mitigated by SPE-fractionation of lipids. Analysis was carried out using untargeted LC-ESI-HRMS (Q Exactive HF) in Full MS/ddMS2 TOP N mode (6), with modifications described in Fig. S5. Results are shown as mean ± SD (n = 3).

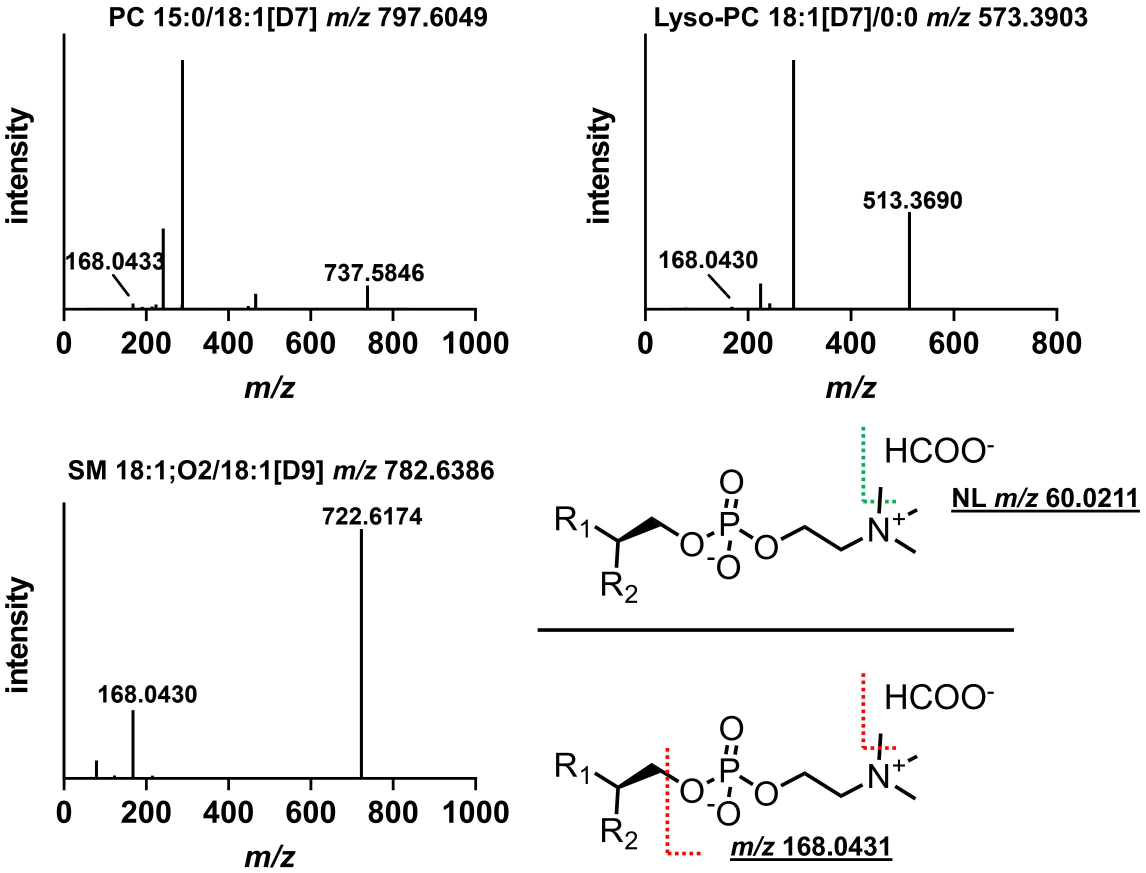

Fig. S9: Characteristic fragment ions of choline-bearing phospholipids in LC-ESI(-)-MS/MS

Shown are MS^2 spectra of $[M+HCOO^-]^-$ ions of PC 15:0/18:1[D7], lyso-PC 15:0/18:1[D7] and SM 18:1;O2/18:1[D9] in ESI(-) mode. The spectra show a similar fragmentation behavior of the choline headgroup for all the compounds: 1) neutral loss (NL) of *m/z* 60.0211 by demethylation and loss of the formate adduct and 2) demethylated $[M-H]^-$ choline phosphate fragment at *m/z* 168.0431 in line with Pi et al. (9). Analysis was carried out using untargeted LC-ESI(-)HRMS (Q Exactive HF Orbitrap) in Full MS/ddMS^2 mode (6) with modifications described in Fig. S5.

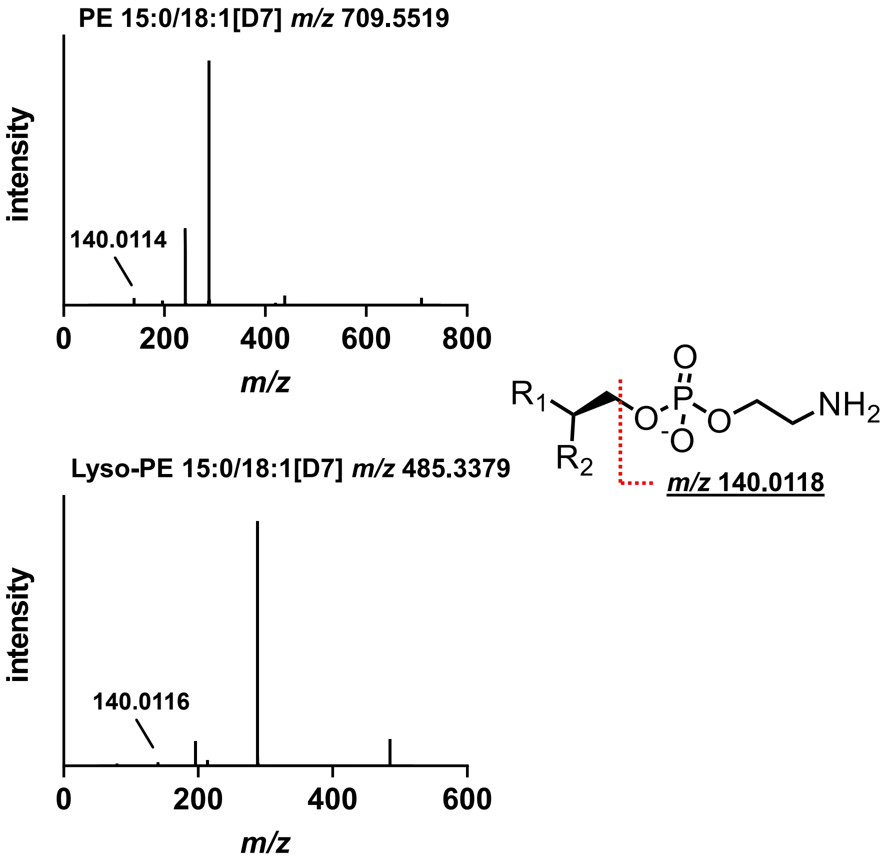

Fig. S10: Characteristic fragment ions of ethanolamine-bearing phospholipids in LC-ESI(-)-MS/MS

Shown are MS^2 spectra of $[M\text{-}H]^-$ ions of PE 15:0/18:1[D7] and lyso-PE 15:0/18:1[D7] in ESI(-) mode. The spectra show similar suggested fragmentation behavior of the ethanolamine headgroup for both compounds: ethanolamine phosphate fragment $[M\text{-}H]^-$ at *m/z* 140.0118 in line with Pi et al. (9). Analysis was carried out using untargeted LC-HRMS (Q Exactive HF Orbitrap) in Full MS/ddMS^2 mode (6) with modifications described in Fig. S5.

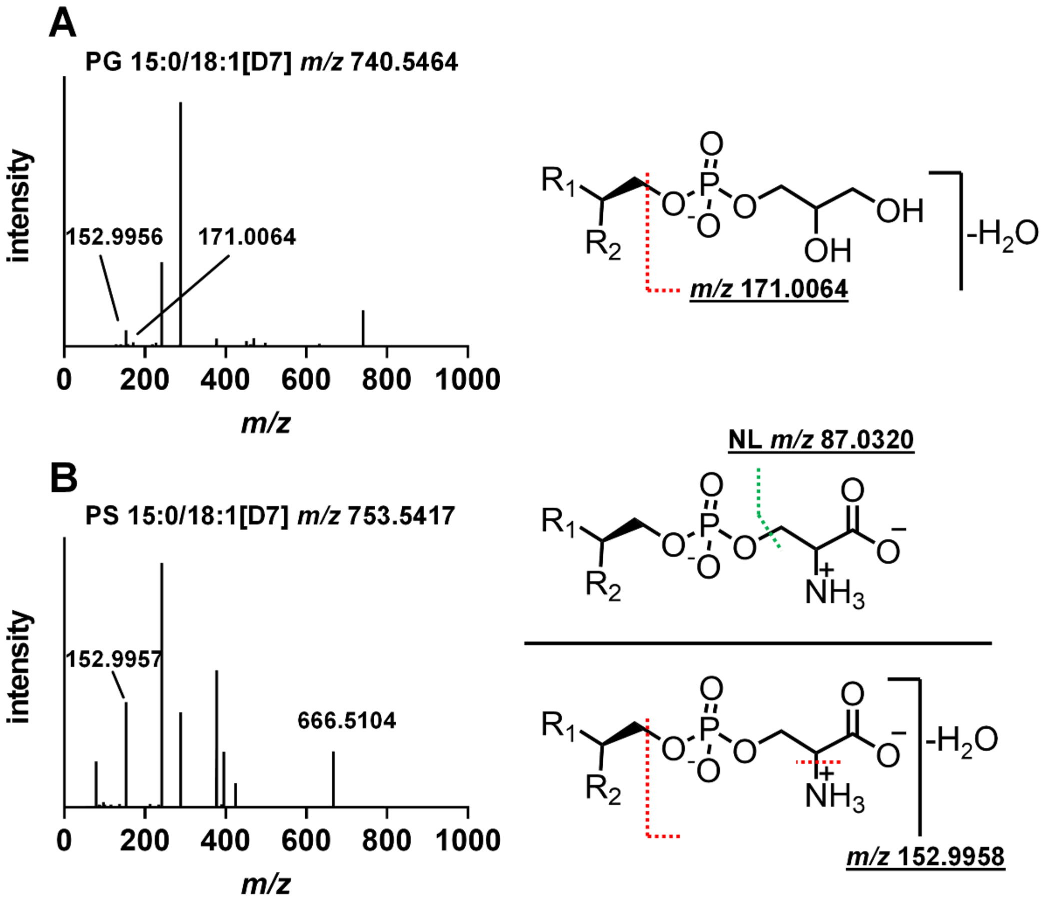

Fig. S11: Characteristic fragment ions of glycerol- and serine-bearing phospholipids in LC-ESI(-)-MS/MS

(A) MS^2 spectrum of PG 15:0/18:1[D7] [M-H]$^-$ in ESI(-) mode. The spectrum shows a characteristic fragment ion of PG: glycerol phosphate fragment [M-H]$^-$ with H_2O loss at *m/z* 171.0064. **(B)** MS^2 spectrum of PS 15:0/18:1[D7] [M-H]$^-$ in ESI(-) mode. The spectrum shows characteristic fragment ions of PS: neutral loss of *m/z* 87.0320 through loss of the serine group in combination with the serine phosphate fragment [M-H]$^-$ after deamination and loss of H_2O at *m/z* 152.9958 in line with Pi et al. (9). Analysis was carried out using untargeted LC-HRMS (Q Exactive HF Orbitrap) in Full MS/ddMS^2 mode (6) with modifications described in Fig. S5.

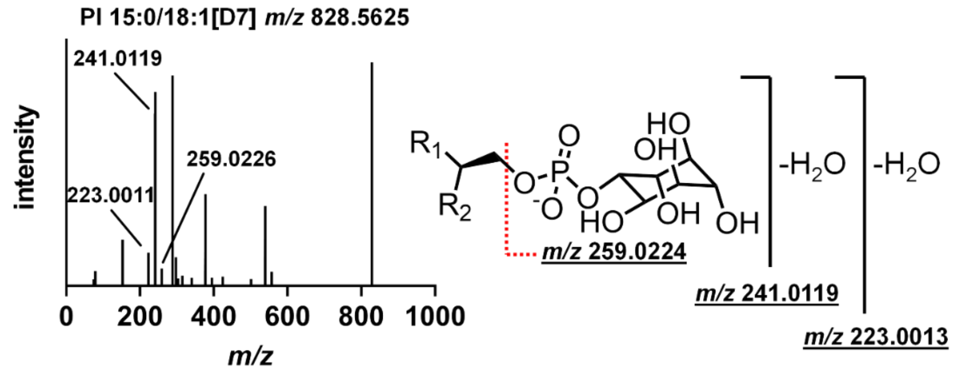

Fig. S12: Characteristic fragment ions of inositol-bearing phospholipids in LC-ESI(-)-MS/MS Shown is a MS^2 spectrum of $[M-H]^-$ ion of PI 15:0/18:1[D7] in ESI(-) mode. The spectrum shows characteristic fragment ions of PI: inositol phosphate fragment $[M-H]^-$ at *m/z* 259.0224 with loss of one H_2O at *m/z* 241.0119 and loss of two H_2O at *m/z* 223.0013 in line with Pi et al. (9). Analysis was carried out using untargeted LC-HRMS (Q Exactive HF Orbitrap) in Full MS/ddMS^2 TOP N mode (6) with modifications described in Fig. S5.

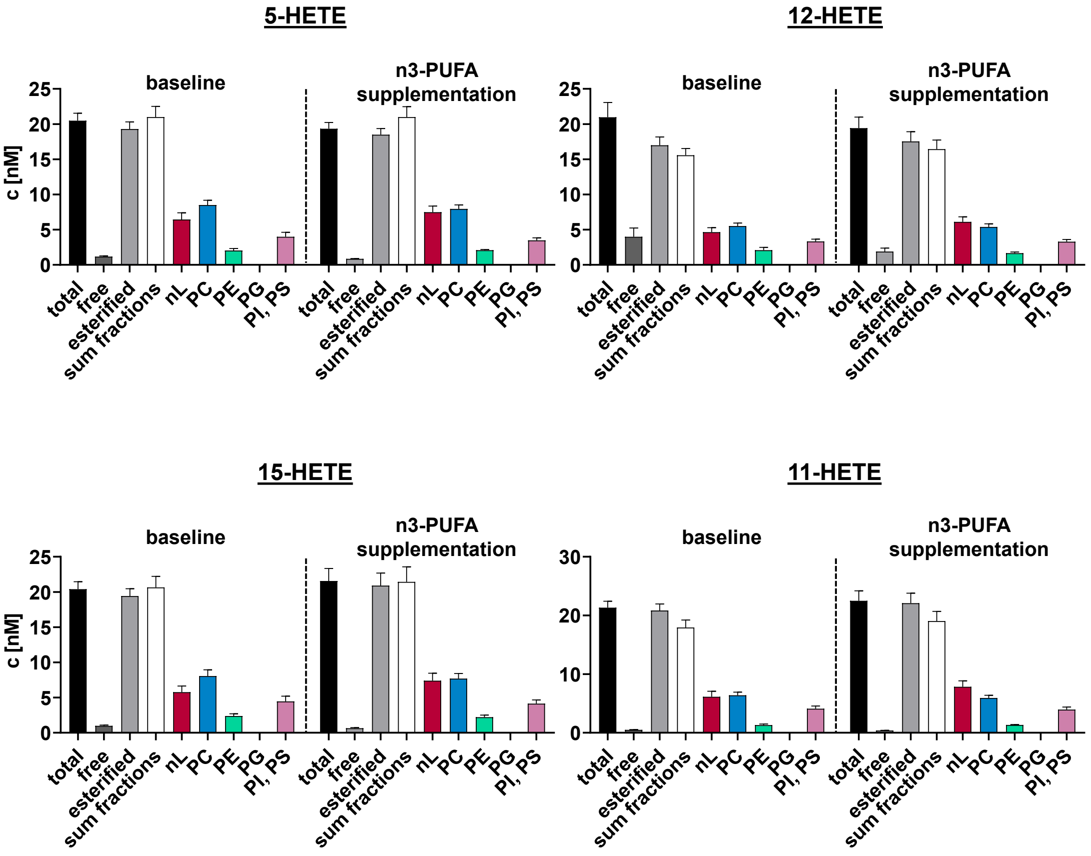

Fig. S13. Concentration and distribution of hydroxy-ARA in human plasma esterified in different lipid classes at baseline and after 12 months of n3-PUFA supplementation Total oxylipins were quantified in the lipid class fractions in plasma of human subjects at baseline and after 12 months of n3-PUFA (1.5 g EPA and 1.8 g DHA/portion; 4 portions per week) supplementation (n = 9). Shown is the concentration ± SEM (n = 9) of free and total hydroxy-ARA. All determined concentrations can be found in Table S10. Analysis was carried out using targeted RP-LC-ESI(-)-MS/MS (3-5).

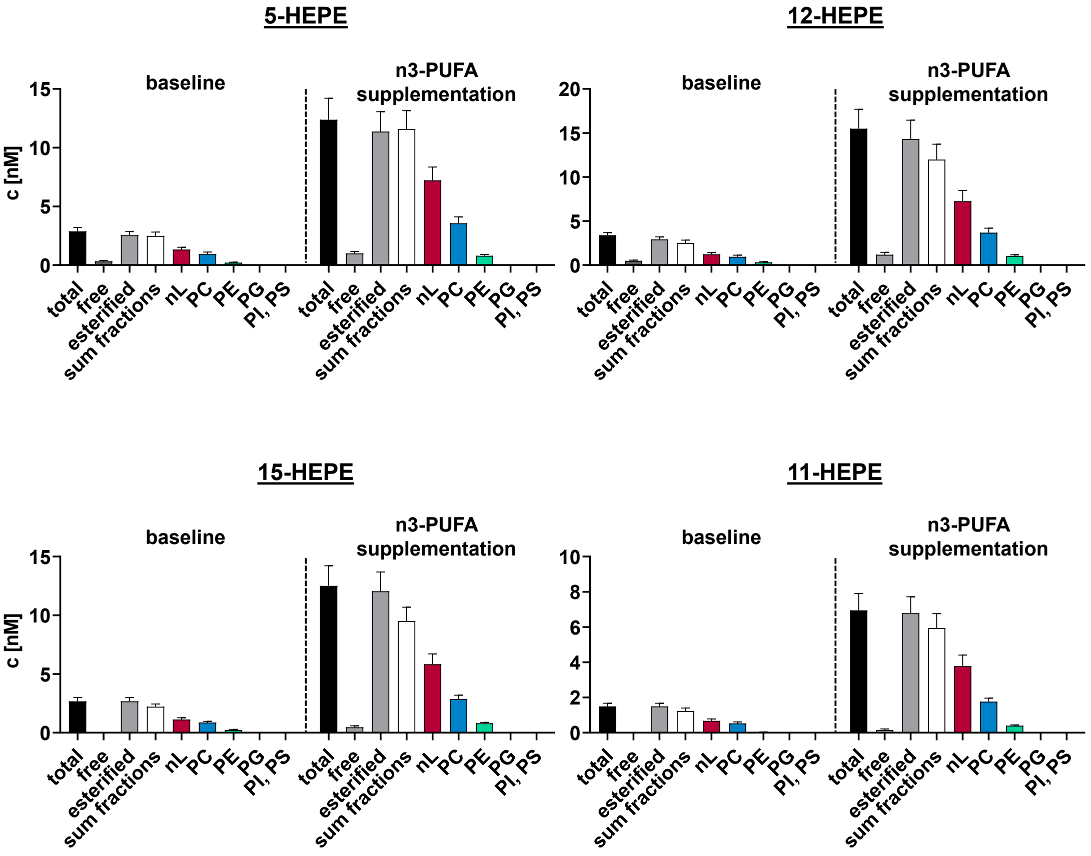

Fig. S14. Concentration and distribution of hydroxy-EPA in human plasma esterified in different lipid classes at baseline and after 12 months of n3-PUFA supplementation Total oxylipins were quantified in the lipid class fractions in plasma of human subjects at baseline and after 12 months of n3-PUFA (1.5 g EPA and 1.8 g DHA/portion; 4 portions per week) supplementation (n = 9). Shown is the concentration ± SEM (n = 9) of free and total hydroxy-EPA. All determined concentrations can be found in Table S10. Analysis was carried out using targeted RP-LC-ESI(-)-MS/MS (3-5).

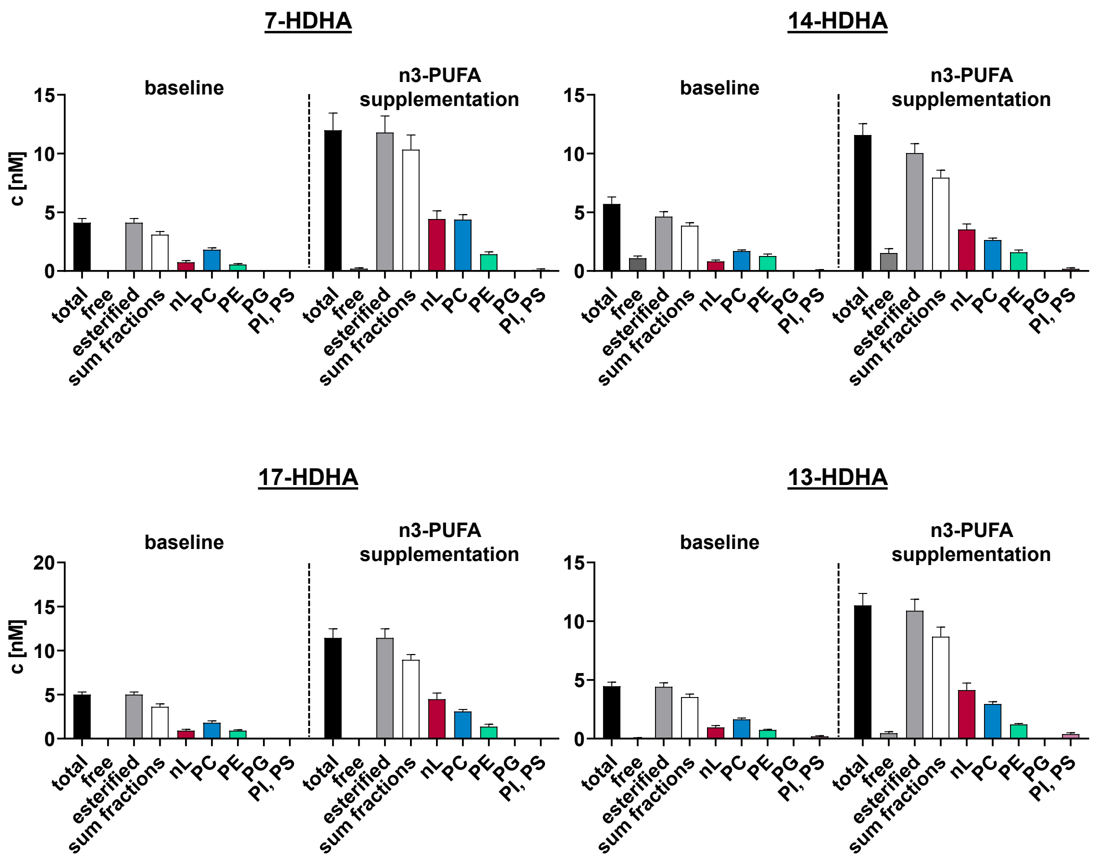

Fig. S15. Concentration and distribution of hydroxy-DHA in human plasma esterified in different lipid classes at baseline and after 12 months of n3-PUFA supplementation Total oxylipins were quantified in the lipid class fractions in plasma of human subjects at baseline and after 12 months of n3-PUFA (1.5 g EPA and 1.8 g DHA/portion; 4 portions per week) supplementation (n = 9). Shown is the concentration ± SEM (n = 9) of free and total hydroxy-DHA. All determined concentrations can be found in Table S10. Analysis was carried out using targeted RP-LC-ESI(-)-MS/MS (3-5).

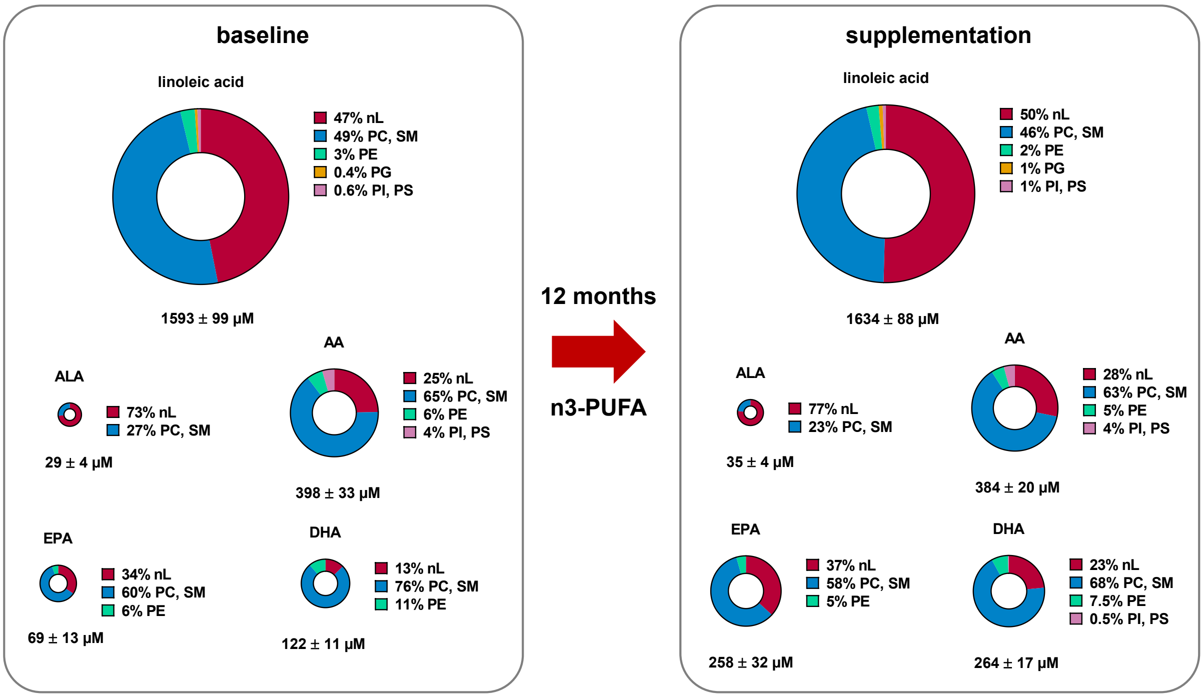

Fig. S16. Change of fatty acids esterified in different lipid classes in human plasma following n3-PUFA supplementation.

Plasma from 9 human subjects was analyzed at baseline and after 12 months of n3-PUFA (1.5 g EPA and 1.8 g DHA/portion; 4 portions per week) supplementation. Results show concentration and relative lipid class distribution of selected FA in plasma at baseline and after n3-PUFA supplementation. EPA increased from 69 ± 13 µM at baseline to 258 ± 32 µM while the distribution pattern remained comparable. DHA increased from 122 ± 11 µM to 263 ± 17 µM and a shift towards an esterification in nLs was observed. All determined concentrations can be found in Table S10. The areas of the circles reflect the relative concentration of the fatty acids between each other. Analysis was carried out using targeted RP-LC-ESI(-)-MS/MS. Shown are the concentrations ± SEM (n = 9) (3-5).

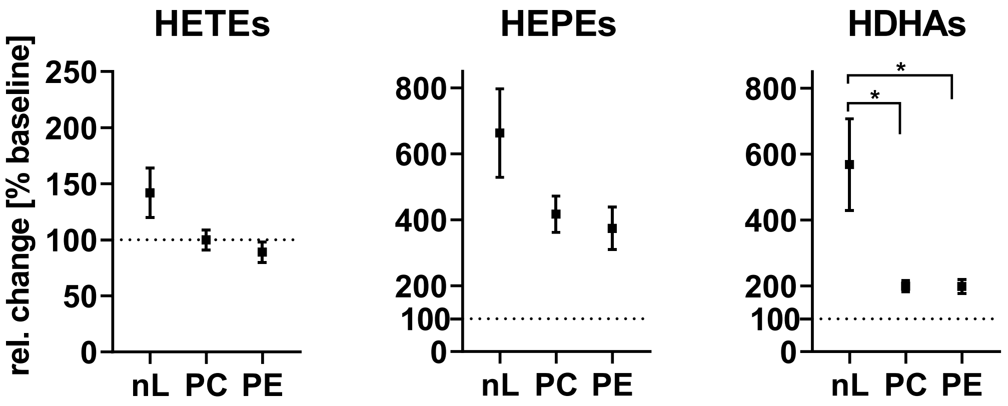

Fig. S17. Changes of hydroxy-PUFA concentration in the lipid classes of plasma following 12 months of n3-PUFA supplementation. Total oxylipins were quantified in the lipid class fractions in plasma of human subjects at baseline and after 12 months of n3-PUFA supplementation (1.5 g EPA and 1.8 g DHA per portion; 4 portions per week). Shown are the relative changes in the concentration of the means of all hydroxy-PUFAs of ARA, EPA, and DHA following n3-PUFA supplementation compared to baseline. Differences in the increase in fraction 1 (nLs) vs. the increase in other fractions were elevated by one-way ANOVA followed by Sidak's multiple comparison test with a statistical significance at $p < 0.05$ indicated by as *. The concentration of the hydroxy-PUFA and the relative changes can be found in the SI (Tables S10 and S11).

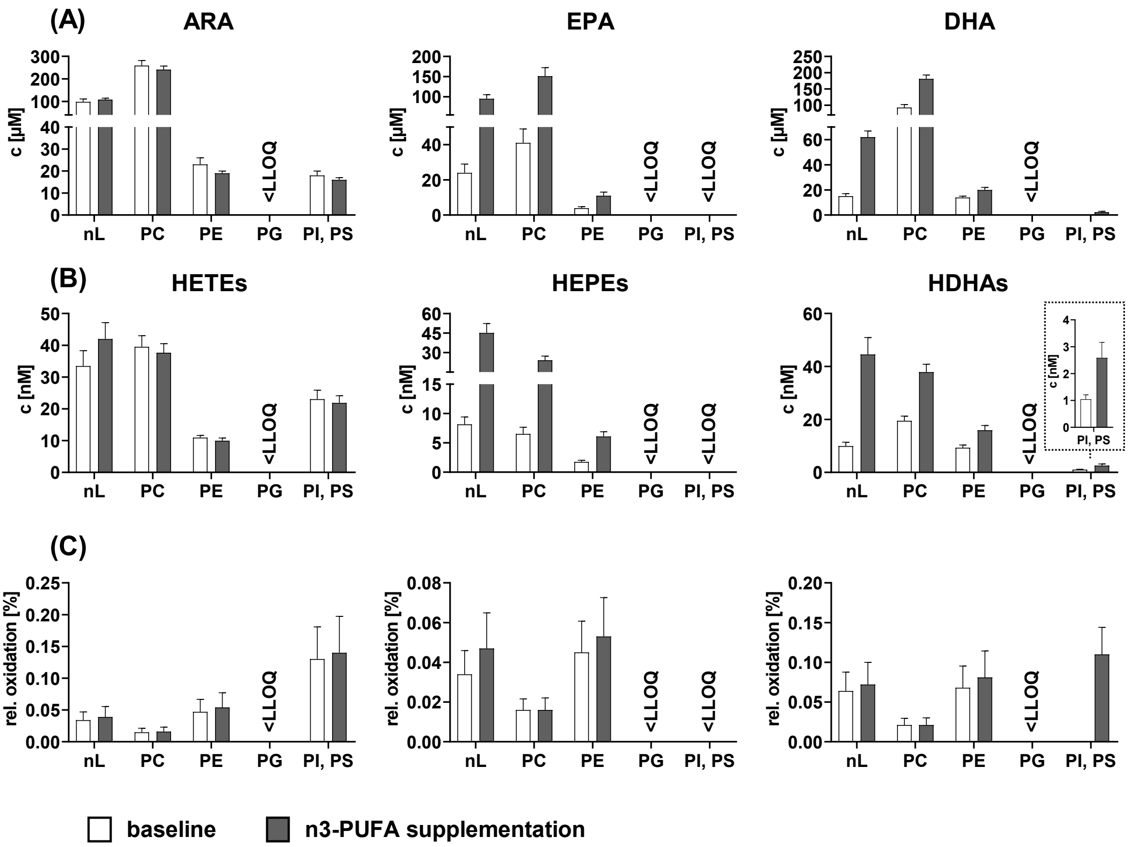

Fig. S18 Comparison of the concentration of ARA, EPA and DHA and the sum of their hydroxy-PUFAs in the lipid fractions of human plasma before and after n3-PUFA supplementation Shown are (A) concentrations of ARA, EPA and DHA and (B) sum of all ARA, EPA and DHA derived hydroxy fatty acids respectively at baseline and following 12 months of n3-PUFA supplementation (1.5 g EPA and 1.8 g DHA per portion; 4 portions per week). (C) Percentage of ratio of sum of hydroxy-PUFAs to precursor PUFA indicating part of the oxidation rate ARA, EPA and DHA. Analysis was carried out using targeted RP-LC-ESI(-)-MS/MS. Shown are the concentrations ± SEM (n = 9) (3-5)

References

1. Browning LM, Walker CG, Mander AP, West AL, Madden J, Gambell JM, et al. Incorporation of eicosapentaenoic and docosahexaenoic acids into lipid pools when given as supplements providing doses equivalent to typical intakes of oily fish. Am J Clin Nutr. 2012;96(4):748-58.

2. Rund KM, Carpanedo L, Lauterbach R, Wermund T, West AL, Wende LM, et al. LC-ESI-HRMS - lipidomics of phospholipids : Characterization of extraction, chromatography and detection parameters. Anal Bioanal Chem. 2024;416(4):925-44.

3. Kutzner L, Rund KM, Ostermann AI, Hartung NM, Galano J-M, Balas L, et al. Development of an Optimized LC-MS Method for the Detection of Specialized Pro-Resolving Mediators in Biological Samples. Frontiers in pharmacology. 2019;10:169.

4. Rund KM, Ostermann AI, Kutzner L, Galano JM, Oger C, Vigor C, et al. Development of an LC-ESI(-)-MS/MS method for the simultaneous quantification of 35 isoprostanes and isofurans derived from the major n3- and n6-PUFAs. Anal Chim Acta. 2018;1037:63-74.

5. Koch E, Mainka M, Dalle C, Ostermann AI, Rund KM, Kutzner L, et al. Stability of oxylipins during plasma generation and long-term storage. Talanta. 2020;217:121074.

6. Carpanedo L, Rund KM, Wende LM, Kampschulte N, Schebb NH. LC-HRMS analysis of phospholipids bearing oxylipins. Analytica Chimica Acta. 2024;1326.

7. Tsugawa H, Cajka T, Kind T, Ma Y, Higgins B, Ikeda K, et al. MS-DIAL: data-independent MS/MS deconvolution for comprehensive metabolome analysis. Nat Methods. 2015;12(6):523-6.

8. Liebisch G, Vizcaino JA, Kofeler H, Trotzmuller M, Griffiths WJ, Schmitz G, et al. Shorthand notation for lipid structures derived from mass spectrometry. J Lipid Res. 2013;54(6):1523-30.

9. Pi J, Wu X, Feng Y. Fragmentation patterns of five types of phospholipids by ultra-high-performance liquid chromatography electrospray ionization quadrupole time-of-flight tandem mass spectrometry. Analytical Methods. 2016;8(6):1319-32.
