# Supplementary material for: Quantification of esterified oxylipins following HILIC-fractionation of lipid classes
Source: J Lipid Res. 2025 Nov 21;67(1):100950. doi: 10.1016/j.jlr.2025.100950 (PMC12799955; doi:10.1016/j.jlr.2025.100950)
Supplement: Oxylipin Method Report [file mmc3.pdf]

# Supplemental data: Details about the oxylipin method

---

# Quantification of esterified oxylipins following HILIC-fractionation of lipid classes

Luca M. Wende[1], Laura Carpanedo[1], Lilli Scholz[1], Nadja Kampschulte[1], Annette L. West[2], Philip C. Calder[2,3], Nils Helge Schebb[1]

[1] Food Chemistry, School of Mathematics and Natural Sciences, University of Wuppertal, 42119 Wuppertal, Germany,

[2] School of Human Development and Health, Faculty of Medicine, University of Southampton, Southampton SO16 6YD, UK

[3] NIHR Southampton Biomedical Research Centre, University Hospital Southampton NHS Foundation Trust and University of Southampton, Southampton SO16 6YD, UK

*corresponding author: Nils Helge Schebb, Food Chemistry, School of Mathematics and Natural Sciences, University of Wuppertal, Gausstrasse 20, 42119 Wuppertal, Germany; Email: nils@schebb-web.de; Phone: +49-202-439-3457

**Table 1: Liquid chromatography**

| | | | |
|---|---|---|---|
| LC system | Agilent Infinity II (Agilent Technologies, Waldbronn, Germany) | | |
| Autosampler | Agilent 1290 Multisampler (G7167B) | | |
| Pump | Agilent 1290 High Speed Pump (G7120A) | | |
| Separation conditions | Reversed-phase | | |
| Column | Zorbax Eclipse Plus C18 (2.1 x 150 mm, 1.8 µm, 9.5 nm) (Agilent Technologies) | | |
| Guard column | Guard column (AJ0-8782) UHPLC C18 (2.1 mm ID) (Phenomenex, Aschaffenburg, Germany) | | |
| Column oven temperature | 40 °C | | |
| Solvents | A: water/B 955 (v/v) + 0.1% acetic acid<br>B: methanol/acetonitrile/acetic acid 800/150/1 (v/v/v) | | |
| Flow rate | 300 µL/min | | |
| Gradient | **Time [min]** | **%A** | **%B** |
| | 0.0 | 78 | 22 |
| | 1.0 | 78 | 22 |
| | 1.5 | 68 | 32 |
| | 10.0 | 48 | 52 |
| | 19.0 | 32 | 68 |
| | 25.1 | 2 | 98 |
| | 27.6 | 2 | 98 |
| | 27.7 | 78 | 22 |
| | 31.1 | 78 | 22 |

**Table 2: Mass spectrometry: instrument and settings**

| | |
|---|---|
| **Instrument** | |
| Mass Spectrometer | Sciex QTRAP 5500 (AB Sciex, Darmstadt, Germany) |
| Ionization mode | ESI(-) |
| **Source parameters** | |
| Curtain gas (CUR) | 50 psi |
| Collision gas (CAD) | high (12) |
| Ion spray voltage (IS) | -4500 kV |
| Temperature (TEM) | 650°C |
| Ion source gas 1 (GS1) | 30 psi |
| Ion source gas 2 (GS2) | 70 psi |
| **MS settings** | |
| Operating mode | scheduled selected reaction monitoring |
| MRM detection window | 45 sec |
| Cycle time | 0.4 sec |
| Data points per peak | 14–18 |
| **Software and libraries** | |
| Analyst 1.6.3. (AB Sciex) | |

**Table 3: MS transitions and MS settings**

| Q1 *m/z* | Q3 *m/z* | retention time (min) | oxylipin[1] | internal standard | DP | EP | CE | CXP |
|---|---|---|---|---|---|---|---|---|
| 293.2 | 171.2 | 16.73 | 9-HOTrE | d4-13-HODE | -85 | -10 | -20 | -8 |
| 293.2 | 195.1 | 17.11 | 13-HOTrE | d4-13-HODE | -90 | -10 | -22 | -8 |
| 317.2 | 259.2 | 17.18 | 18-HEPE | d8-15-HETE | -75 | -10 | -15 | -7 |
| 317.2 | 219.2 | 17.92 | 15-HEPE | d8-15-HETE | -80 | -10 | -18 | -10 |
| 317.2 | 167.0 | 18.03 | 11-HEPE | d8-15-HETE | -70 | -10 | -19 | -8 |
| 295.2 | 223.0 | 18.05 | 15-HODE | d4-13-HODE | -95 | -10 | -24 | -9 |
| 317.2 | 155.2 | 18.26 | 8-HEPE | d8-15-HETE | -80 | -10 | -18 | -8 |
| 317.2 | 179.2 | 18.46 | 12-HEPE | d8-15-HETE | -85 | -10 | -18 | -8 |
| 317.2 | 167.0 | 18.63 | 9-HEPE | d8-15-HETE | -70 | -10 | -17 | -8 |
| 317.2 | 115.1 | 18.98 | 5-HEPE | d8-15-HETE | -80 | -10 | -18 | -6 |
| 295.2 | 195.2 | 19.19 | 13-HODE | d4-9-HODE | -100 | -10 | -24 | -9 |
| 295.2 | 171.1 | 19.30 | 9-HODE | d4-9-HODE | -100 | -10 | -24 | -7 |
| 343.2 | 241.2 | 19.59 | 20-HDHA | d8-15-HETE | -75 | -10 | -17 | -7 |
| 319.2 | 219.2 | 19.96 | 15-HETE | d8-15-HETE | -80 | -10 | -18 | -8 |
| 343.2 | 233.2 | 20.15 | 16-HDHA | d8-15-HETE | -75 | -10 | -17 | -7 |
| 343.2 | 245.1 | 20.30 | 17-HDHA | d8-15-HETE | -80 | -10 | -18 | -6 |
| 343.2 | 193.2 | 20.48 | 13-HDHA | d8-12-HETE | -75 | -10 | -17 | -7 |
| 319.2 | 167.2 | 20.54 | 11-HETE | d8-12-HETE | -80 | -10 | -21 | -7 |
| 343.2 | 153.2 | 20.74 | 10-HDHA | d8-12-HETE | -70 | -10 | -19 | -7 |
| 343.2 | 205.2 | 20.74 | 14-HDHA | d8-12-HETE | -70 | -10 | -17 | -7 |
| 319.2 | 155.2 | 20.94 | 8-HETE | d8-12-HETE | -80 | -10 | -20 | -6 |
| 319.2 | 179.2 | 20.98 | 12-HETE | d8-12-HETE | -80 | -10 | -19 | -8 |
| 343.2 | 121.1 | 21.06 | 11-HDHA | d8-5-HETE | -70 | -10 | -18 | -7 |
| 343.2 | 141.2 | 21.20 | 7-HDHA | d8-5-HETE | -75 | -10 | -17 | -7 |
| 319.2 | 167.2 | 21.29 | 9-HETE | d8-5-HETE | -80 | -10 | -21 | -7 |
| 321.2 | 221.2 | 21.35 | 15-HETrE | d8-5-HETE | -90 | -10 | -21 | -10 |
| 343.2 | 189.2 | 21.38 | 8-HDHA | d8-5-HETE | -70 | -10 | -17 | -7 |
| 319.2 | 115.2 | 21.57 | 5-HETE | d8-5-HETE | -80 | -10 | -19 | -7 |
| 321.2 | 157.1 | 21.70 | 8-HETrE | d8-5-HETE | -85 | -10 | -22 | -9 |
| 321.0 | 181.0 | 21.90 | 12-HETrE | d8-5-HETE | -85 | -10 | -24 | -10 |
| 343.2 | 101.1 | 22.07 | 4-HDHA | d8-5-HETE | -75 | -10 | -17 | -7 |
| 321.2 | 115.1 | 23.41 | 5-HETrE | d8-5-HETE | -90 | -10 | -17 | -9 |

[1] Oxylipins were identified in samples based on (relative) retention time and MRM transition.

**Definition of LLOQ and LOD**

The limit of detection (LOD) and lower limit of quantification (LLOQ) were determined as described (1). The LOD was set to the concentration of the lowest standard injected yielding a signal-to-noise-ratio (S/N) ≥ 3. The LLOQ was set to the concentration of the lowest calibration standard yielding an S/N ≥ 5 and an accuracy within 100 ± 20% using the linear calibration function.

**Table 4: LLOQ [nM, injected solution, injection volume 5 µL] (S/N ≥ 5) of quantified oxylipins determined in matrix free standard solutions**

| oxylipin | LLOQ [nM] |
|---|---|
| 9-HOTrE | 0.25 |
| 13-HOTrE | 0.5 |
| 18-HEPE | 0.1 |
| 15-HEPE | 0.1 |
| 11-HEPE | 0.062 |
| 15-HODE | 0.18 |
| 8-HEPE | 0.06 |
| 12-HEPE | 0.1 |
| 9-HEPE | 0.25 |
| 5-HEPE | 0.06 |
| 13-HODE | 0.25 |
| 9-HODE | 0.35 |
| 20-HDHA | 0.25 |
| 15-HETE | 0.22 |
| 16-HDHA | 0.25 |
| 17-HDHA | 0.9 |
| 13-HDHA | 0.1 |
| 11-HETE | 0.044 |
| 10-HDHA | 0.05 |
| 14-HDHA | 0.14 |
| 8-HETE | 0.23 |
| 12-HETE | 0.25 |
| 11-HDHA | 0.25 |
| 7-HDHA | 0.1 |
| 9-HETE | 0.4 |
| 15-HETrE | 0.1 |
| 8-HDHA | 0.1 |
| 5-HETE | 0.035 |
| 8-HETrE | 0.5 |
| 12-HETrE | 0.25 |
| 4-HDHA | 0.1 |
| 5-HETrE | 0.025 |

**Sample preparation**

Details on sample preparation, including an SOP can be found in (1-5). For the analysis of free (non-esterified) oxylipins, internal standards (deuterium-labeled oxylipins [each 1 pmol/500 µL plasma/~2 mg cell protein]) and additives (10 µL 0.2 mg/mL butylated hydroxytoluene (BHT), 100 µM indomethacin and 100 µM *trans*-4-[4-(3-adamantan-1-yl-ureido)-cyclohexyloxy]-benzoic acid (t-AUCB) in methanol) were added to 500 µL freshly thawed plasma or 500 µL sonicated cell suspension (~2 mg cell protein) and samples were mixed. The proteins were precipitated by addition of 1400 µL ice cold MeOH and storage at -80°C for at least 30 min. After centrifugation (10 min, 4°C, 20,000×g) the supernatant was evaporated using a vacuum concentrator (1 mbar, 30°C; Christ, Osterode am Harz, Germany) to a volume less than 1 mL and transferred onto the preconditioned SPE cartridges.
For the analysis of total oxylipins, samples were saponified as described (3, 6). In brief, internal standards (deuterium-labeled oxylipins [each 1 pmol/100 µL plasma/~400 µg cell protein]) and additives (10 µL 0.2 mg/mL BHT, 100 µM indomethacin and 100 µM t-AUCB in methanol) were added to 100 µL freshly thawed plasma or 100 µL sonicated cell suspension (~400 µg cell protein) and samples were mixed. 400 µL ice cold iso-propanol (-30°C) were added, and samples were frozen at -80°C for at least 30 min, and subsequently centrifuged (4°C, 10 min, 20 000×g). The supernatant was hydrolyzed at 60 C for 30 min using 100 µL 0.6 M KOH in MeOH/water (75/25, v/v). Following hydrolysis, samples were cooled and neutralized with ~20 µL 25% aqueous acetic acid followed by the transfer onto the preconditioned SPE cartridge.

Oxylipins were extracted using Oasis MAX extraction cartridges (60 mg, 3 mL; Waters, Eschborn, Germany) The SPE cartridges were preconditioned with one cartridge volume of each, ethyl acetate/*n*-hexane (75/25, v/v) containing 1% acetic acid, MeOH and 0.1 M disodium hydrogen phosphate adjusted to pH 6.0 with acetic acid in water/MeOH (95/5, v/v). 2 mL 0.1 M aqueous disodium hydrogen phosphate buffer (pH 6.0) were added together with the sample onto the preconditioned cartridge. The cartridge was washed with 3 mL water and 3 mL water/MeOH (50/50, v/v) and dried under vacuum (200 mbar) for 30 s. The analytes were eluted with 2.0 mL of 75/25 (v/v) ethyl acetate/*n*-hexane with 1% acetic acid in glass tubes containing 6 µL of 30% glycerol in MeOH. The solvent was evaporated using a vacuum concentrator (1 mbar, 30°C, 45-60 min; Christ, Osterode am Harz, Germany) and samples were resuspended in 50 µL MeOH containing 40 nM of 1-(1-(ethylsulfonyl)piperidin-4-yl)-3-(4-(trifluoromethoxy)phenyl)urea as secondary internal standard for the calculation of the recovery of the IS, centrifuged (10 min, 4°C, 20,000×g) and analyzed by LC-MS.

A pool of human plasma from healthy subjects was used as QC sample, and IS recovery, accuracy and precision of the quantified oxylipins were evaluated in each sample batch.

**Table 5: Recovery of internal standards in human plasma samples**

| internal standard | recovery [%] (Mean ± SD; n=50) |
|---|---|
| d4-9-HODE | 87 ± 9 |
| d4-13-HODE | 83 ± 10 |
| d8-5-HETE | 83 ± 10 |
| d8-12-HETE | 82 ± 13 |
| d8-15-HETE | 83 ± 10 |

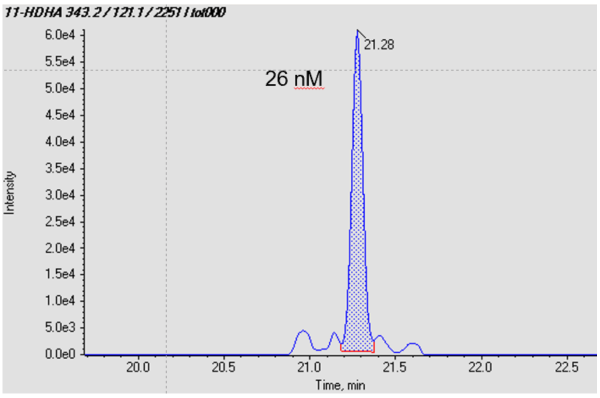
11-HDHA 343.2 / 121.1 / 2251 I tot000
26 nM
21.28
Intensity
Time, min

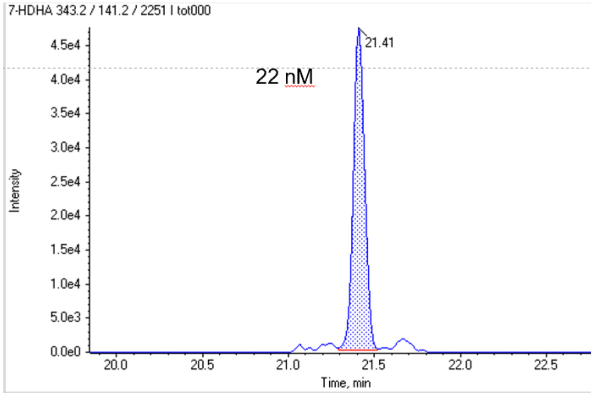
7-HDHA 343.2 / 141.2 / 2251 I tot000
22 nM
21.41
Intensity
Time, min

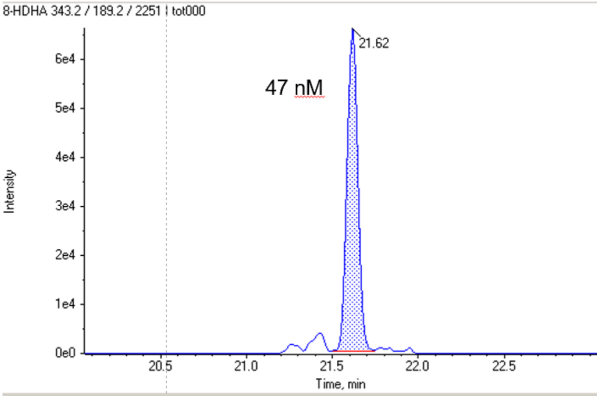
8-HDHA 343.2 / 189.2 / 2251 I tot000
47 nM
21.62
Intensity
Time, min

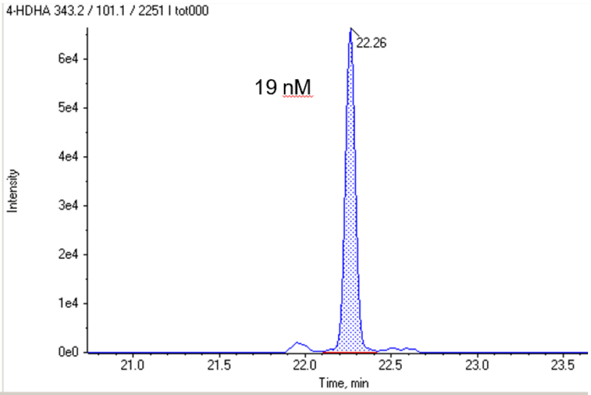
4-HDHA 343.2 / 101.1 / 2251 I tot000
19 nM
22.26
Intensity
Time, min

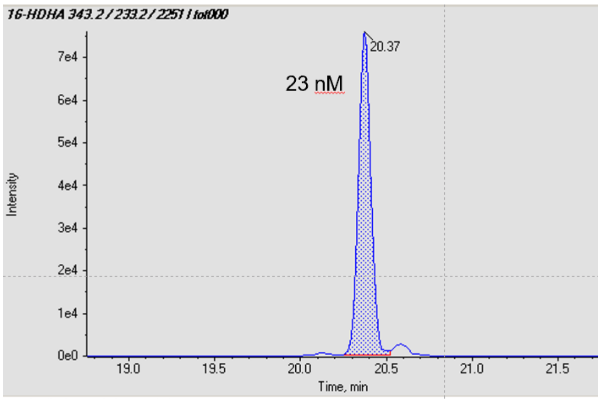
16-HDHA 343.2 / 233.2 / 2251 I tot000
23 nM
20.37
Intensity
Time, min

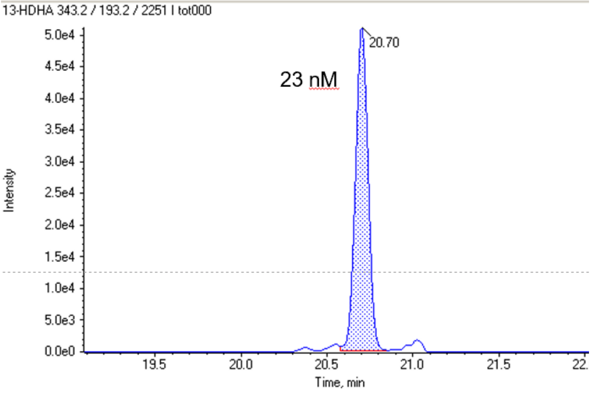
13-HDHA 343.2 / 193.2 / 2251 I tot000
23 nM
20.70
Intensity
Time, min

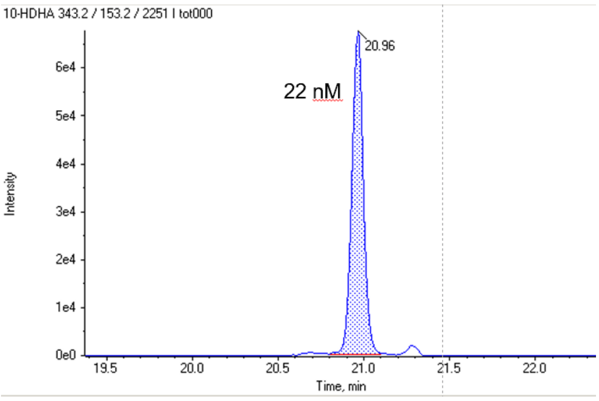
10-HDHA 343.2 / 153.2 / 2251 I tot000
22 nM
20.96
Intensity
Time, min

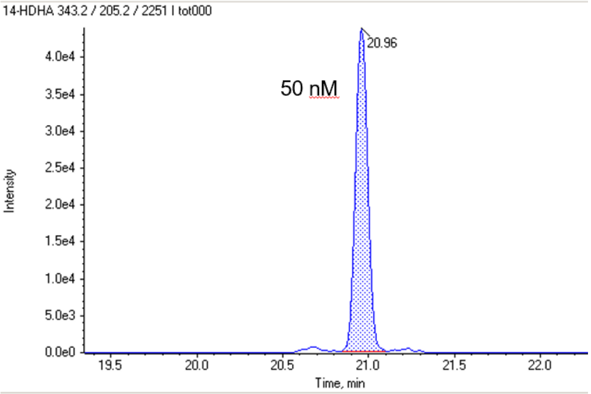
14-HDHA 343.2 / 205.2 / 2251 I tot000
50 nM
20.96
Intensity
Time, min

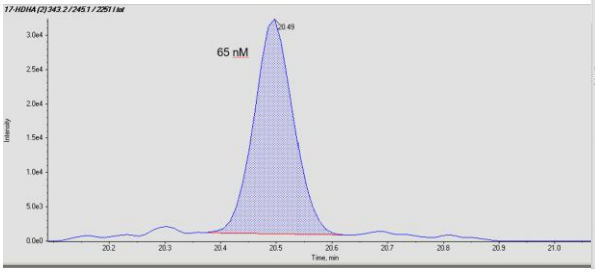
65 nM
20.49
Time, min

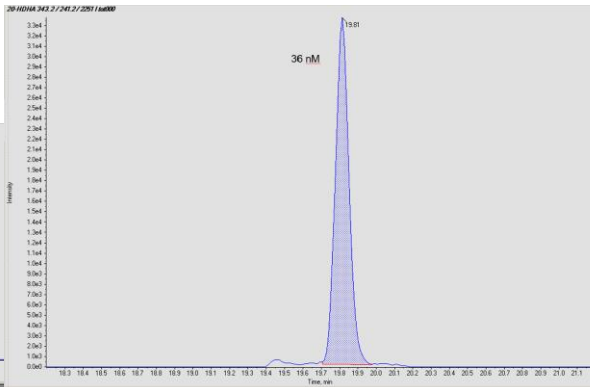
36 nM

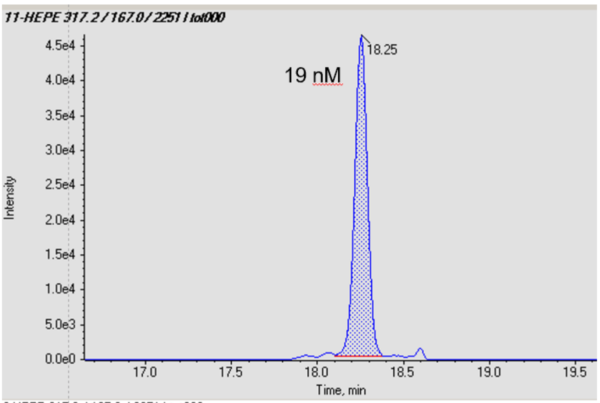
11-HEPE 317.2 / 167.0 / 2251 I tot000
19 nM
18.25
Intensity
Time, min

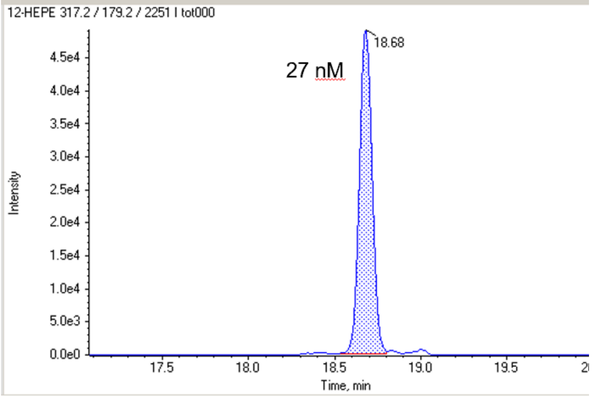
12-HEPE 317.2 / 179.2 / 2251 I tot000
27 nM
18.68
Intensity
Time, min

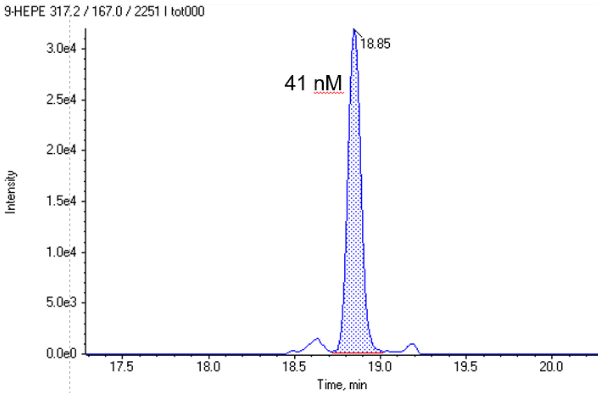
9-HEPE 317.2 / 167.0 / 2251 I tot000
41 nM
18.85
Intensity
Time, min

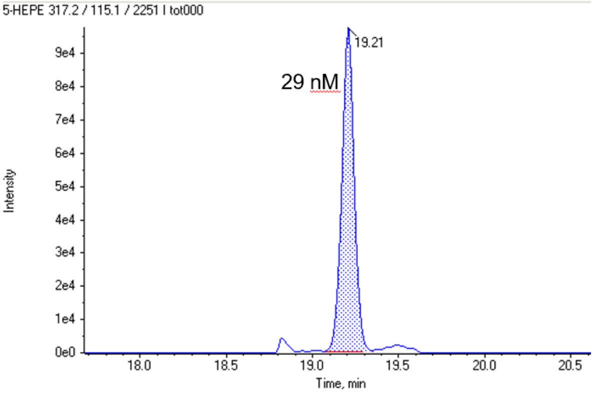
5-HEPE 317.2 / 115.1 / 2251 I tot000
29 nM
19.21
Intensity
Time, min

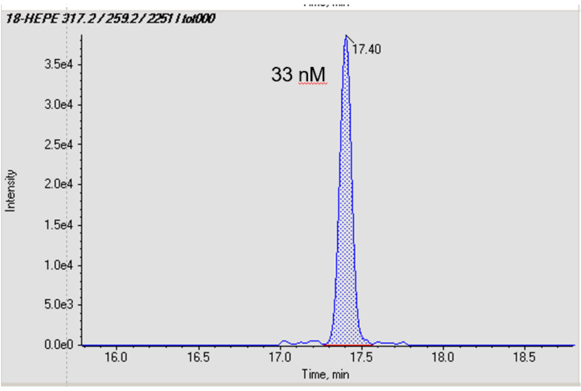
18-HEPE 317.2 / 259.2 / 2251 I tot000
33 nM
17.40
Intensity
Time, min

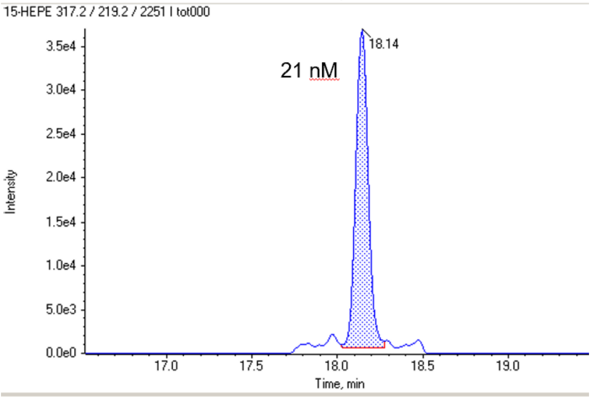
15-HEPE 317.2 / 219.2 / 2251 I tot000
21 nM
18.14
Intensity
Time, min

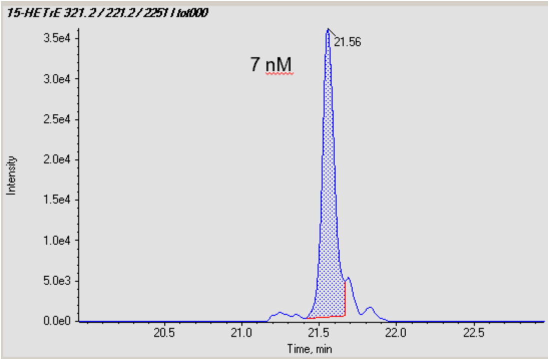
15-HETrE 321.2 / 221.2 / 2251 I tot000
7 nM
21.56
Intensity
Time, min

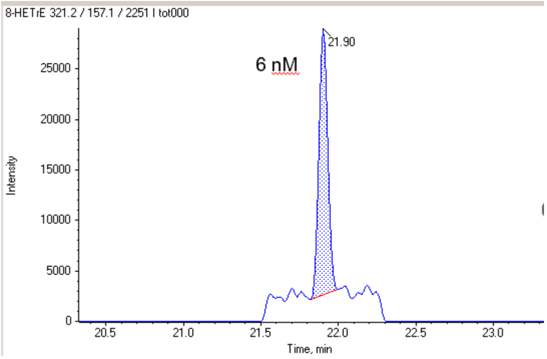
8-HETrE 321.2 / 157.1 / 2251 I tot000
6 nM
21.90
Intensity
Time, min

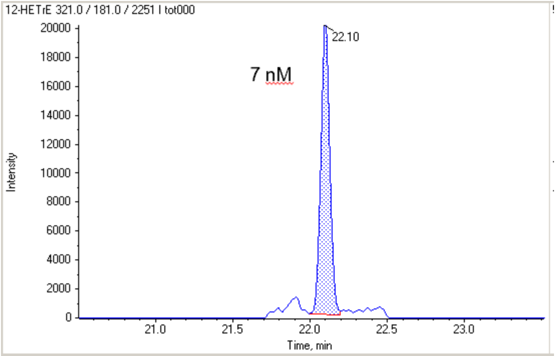
12-HETrE 321.0 / 181.0 / 2251 I tot000
7 nM
22.10
Intensity
Time, min

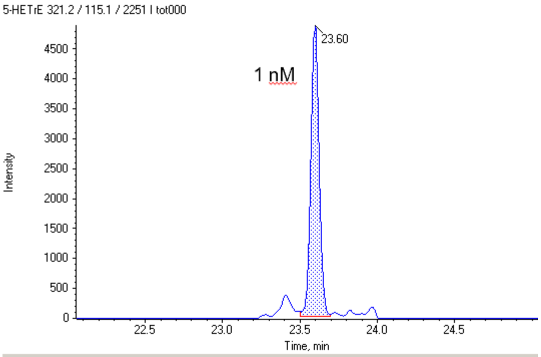
5-HETrE 321.2 / 115.1 / 2251 I tot000
1 nM
23.60
Intensity
Time, min

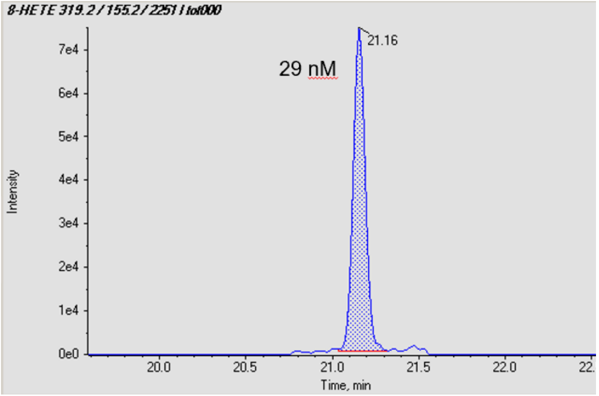
8-HETE 319.2 / 155.2 / 2251 I tot000
29 nM
21.16
Intensity
Time, min

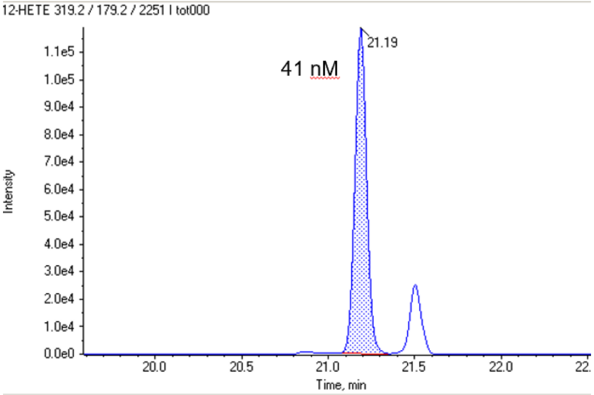
12-HETE 319.2 / 179.2 / 2251 I tot000
41 nM
21.19
Intensity
Time, min

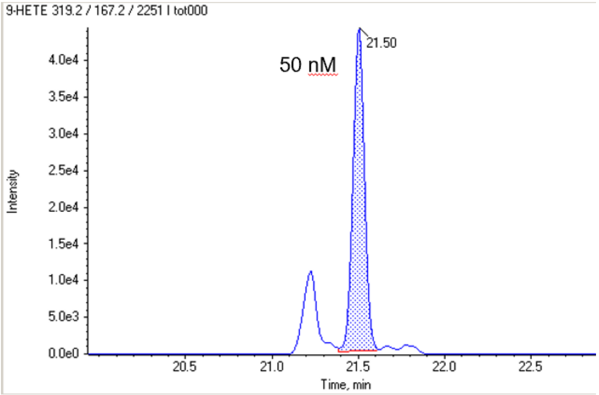
9-HETE 319.2 / 167.2 / 2251 I tot000
50 nM
21.50
Intensity
Time, min

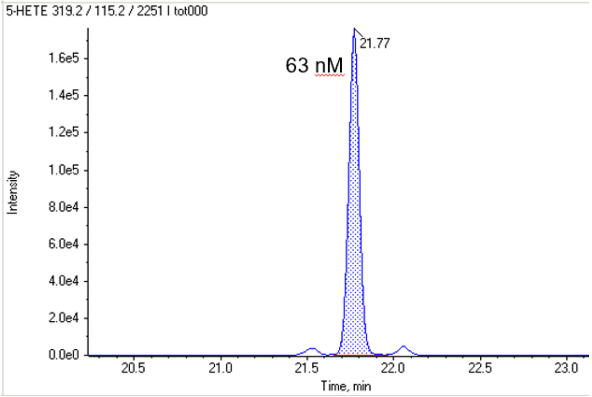
5-HETE 319.2 / 115.2 / 2251 I tot000
63 nM
21.77
Intensity
Time, min

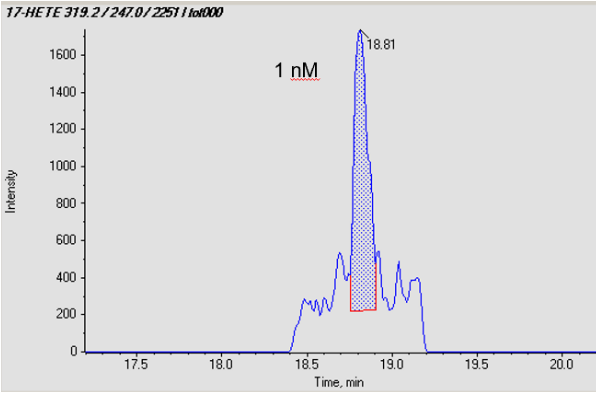
17-HETE 319.2 / 247.0 / 2251 I tot000
1 nM
18.81
Intensity
Time, min

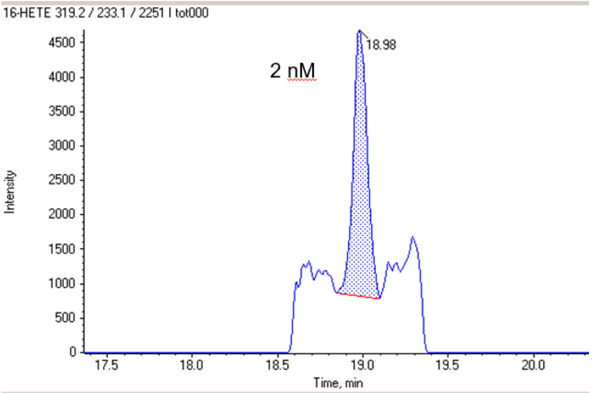
16-HETE 319.2 / 233.1 / 2251 I tot000
2 nM
18.98
Intensity
Time, min

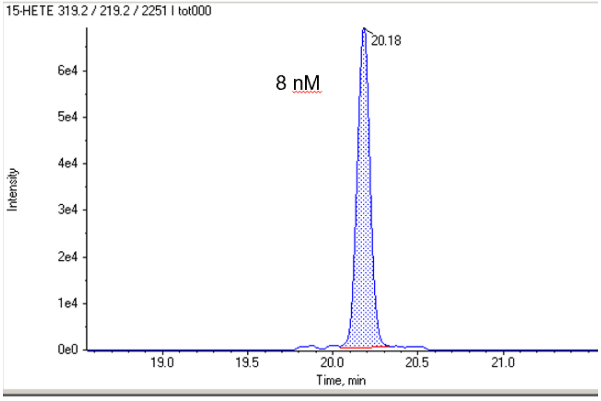
15-HETE 319.2 / 219.2 / 2251 I tot000
8 nM
20.18
Intensity
Time, min

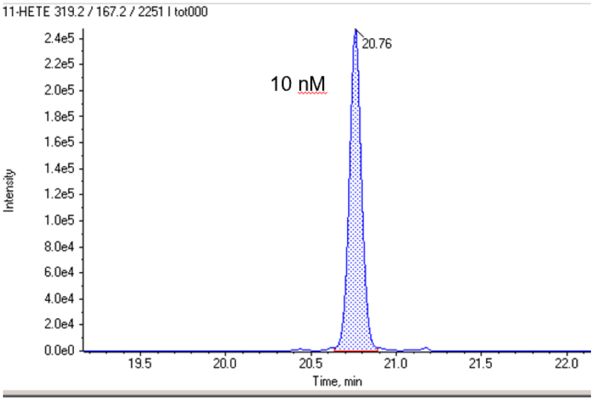
11-HETE 319.2 / 167.2 / 2251 I tot000
10 nM
20.76
Intensity
Time, min

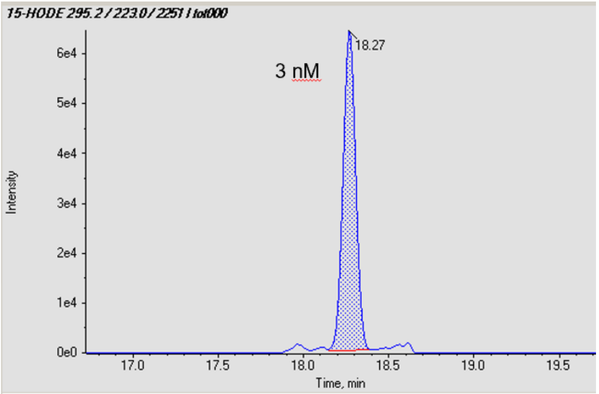
15-HODE 295.2 / 223.0 / 2251 I tot000
3 nM
18.27
Intensity
Time, min

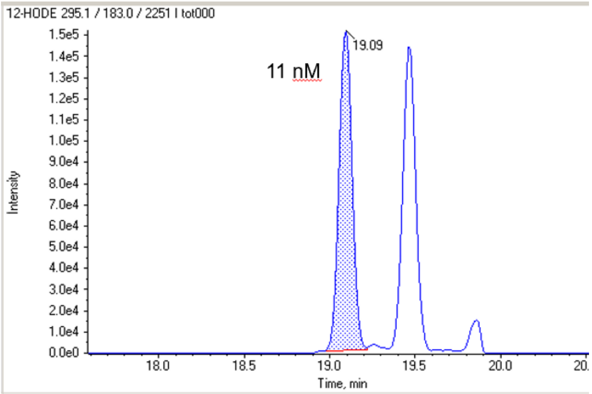
12-HODE 295.1 / 183.0 / 2251 I tot000
11 nM
19.09
Intensity
Time, min

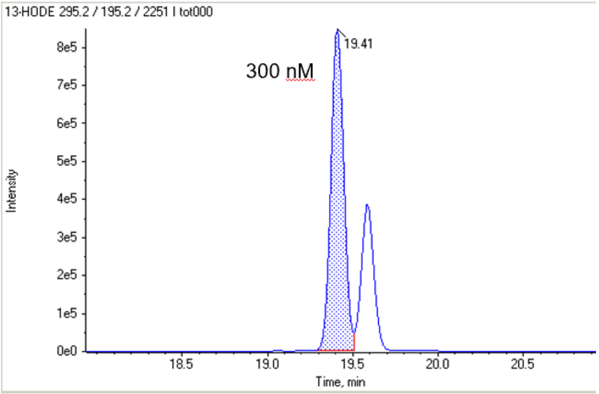
13-HODE 295.2 / 195.2 / 2251 I tot000
300 nM
19.41
Intensity
Time, min

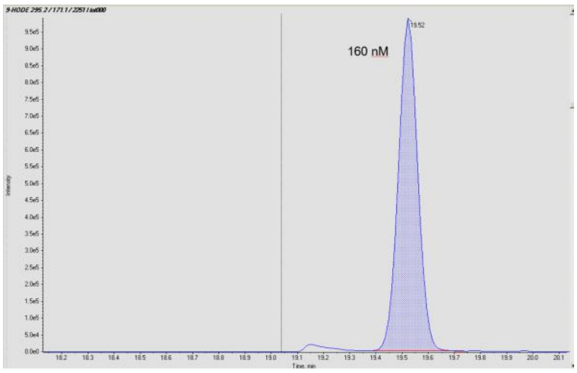
160 nM

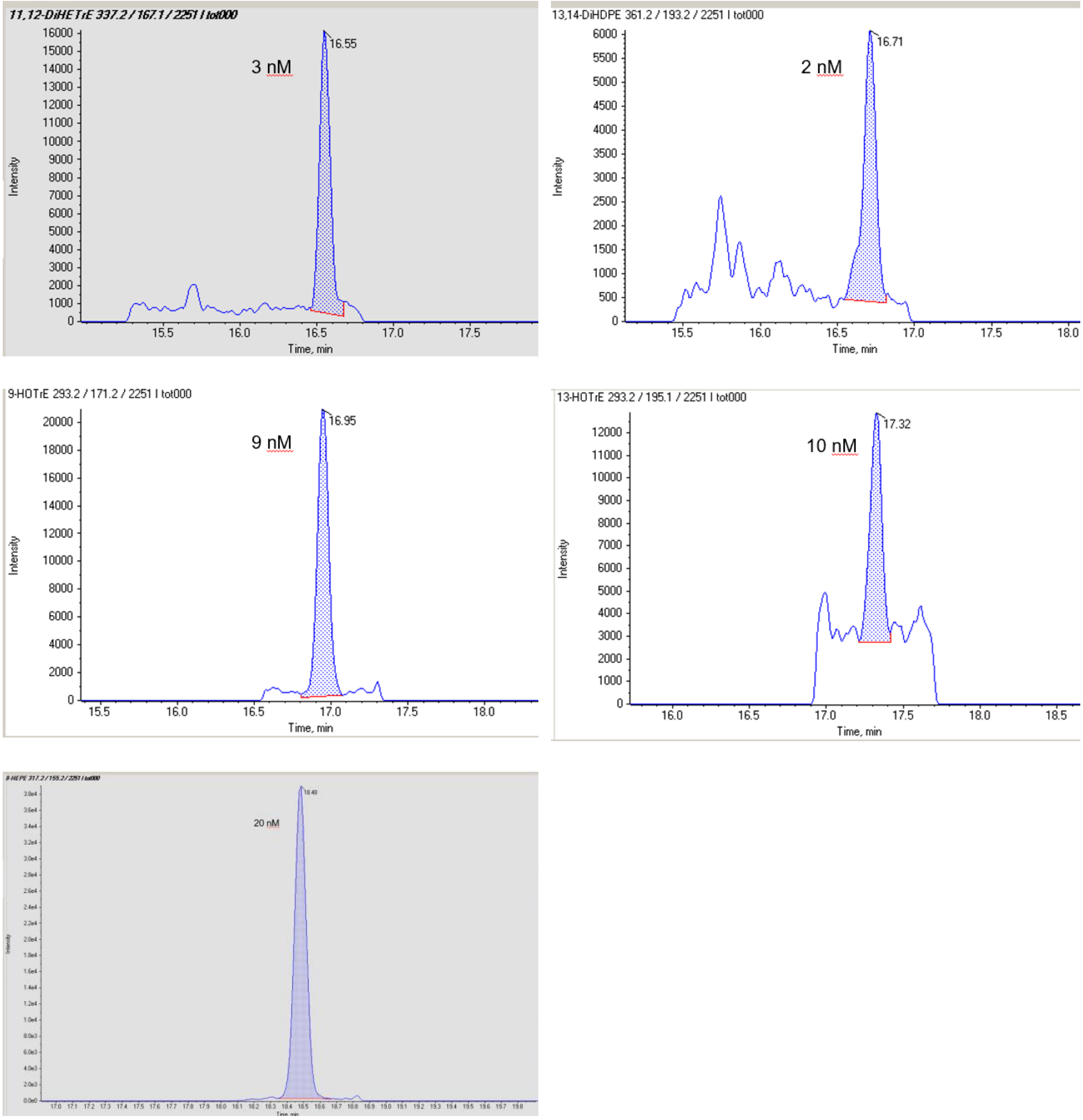


**Figure 1: Chromatograms of oxylipin transitions in representative human plasma extracts of the study and quantified concentrations in the injected solutions (SPE extracts).**

**References**


1. Kutzner L, Rund KM, Ostermann AI, Hartung NM, Galano J-M, Balas L, et al. Development of an Optimized LC-MS Method for the Detection of Specialized Pro-Resolving Mediators in Biological Samples. Frontiers in Pharmacology. 2019;Volume 10 - 2019.

2. Koch E, Mainka M, Dalle C, Ostermann AI, Rund KM, Kutzner L, et al. Stability of oxylipins during plasma generation and long-term storage. Talanta. 2020;217:121074.

3. Mainka M, Dalle C, Pétéra M, Dalloux-Chioccioli J, Kampschulte N, Ostermann AI, et al. Harmonized procedures lead to comparable quantification of total oxylipins across laboratories. Journal of Lipid Research. 2020;61(11):1424-36.

4. Rund KM, Ostermann AI, Kutzner L, Galano J-M, Oger C, Vigor C, et al. Development of an LC-ESI(-)-MS/MS method for the simultaneous quantification of 35 isoprostanes and isofurans derived from the major n3- and n6-PUFAs. Analytica Chimica Acta. 2018;1037:63-74.

5. Rund KM, Schebb NH. Quantitative Analysis of Eicosanoids and Other Oxylipins. In: Ivanisevic J, Giera M, editors. A Practical Guide to Metabolomics Applications in Health and Disease: From Samples to Insights into Metabolism. Cham: Springer International Publishing; 2023. p. 343-69.

6. Ostermann AI, Koch E, Rund KM, Kutzner L, Mainka M, Schebb NH. Targeting esterified oxylipins by LC–MS - Effect of sample preparation on oxylipin pattern. Prostaglandins & Other Lipid Mediators. 2020;146:106384.
